# Pattern Selection in Network of Coupled Multi-Scroll Attractors

**DOI:** 10.1371/journal.pone.0154282

**Published:** 2016-04-27

**Authors:** Fan Li, Jun Ma

**Affiliations:** 1 Wuhan Institute of Physics and Mathematics, Chinese Academy of Sciences, Wuhan, 430071, China; 2 University of Chinese Academy of Sciences, Beijing, 100049, China; 3 Department of Physics, Lanzhou University of Technology, Lanzhou, 730050, China; Universidad Rey Juan Carlos, SPAIN

## Abstract

Multi-scroll chaotic attractor makes the oscillator become more complex in dynamic behaviors. The collective behaviors of coupled oscillators with multi-scroll attractors are investigated in the regular network in two-dimensional array, which the local kinetics is described by an improved Chua circuit. A feasible scheme of negative feedback with diversity is imposed on the network to stabilize the spatial patterns. Firstly, the Chua circuit is improved by replacing the nonlinear term with Sine function to generate infinite aquariums so that multi-scroll chaotic attractors could be generated under appropriate parameters, which could be detected by calculating the Lyapunov exponent in the parameter region. Furthermore, negative feedback with different gains (*D*_1_, *D*_2_) is imposed on the local square center area A_2_ and outer area A_1_ of the network, it is found that spiral wave, target wave could be developed in the network under appropriate feedback gain with diversity and size of controlled area. Particularly, homogeneous state could be reached after synchronization by selecting appropriate feedback gain and controlled size in the network. Finally, the distribution for statistical factors of synchronization is calculated in the two-parameter space to understand the transition of pattern region. It is found that developed spiral waves, target waves often are associated with smaller factor of synchronization. These results show that emergence of sustained spiral wave and continuous target wave could be effective for further suppression of spatiotemporal chaos in network by generating stable pacemaker completely.

## Introduction

Patterns formation and control in spatiotemporal systems have been extensively investigated in the last decades. It is confirmed that various patterns could be observed in physical, chemical, and biological systems, and it is believed that numerical investigation could be feasible to explore some main properties of spatial patterns in dynamical systems. Spiral wave is a class of spatiotemporal pattern, it could be found in the oscillatory and excitable media, such as cardiac tissue [1] and CO oxidation on Pt(110) surface [2]. It has been confirmed that there are involvements of spiral waves in both atrial and ventricular [3–5]. The sudden cardiac death resulting from ventricular fibrillation is due to the fragment or breakup of the spiral wave and the potential mechanism for breakup of spiral wave has been detected [6]. Target wave could be generated by the concentric waves that are periodically emitted from a small central region [7], and is regarded as the generic for dissipative systems far from equilibrium. Transition of spiral wave, target wave and broken patterns can be induced in possible schemes [8–10].

In general way, the reaction-diffusion system [11–16], coupled oscillators with array type [17], network of coupled neurons (Hodgkin-Huxley, Hindmarch-Rose, Morris-Lecar) [18–24] are suitable for generating spiral wave, target wave and even spatiotemporal chaos in numerical studies. For example, He et al. [17] reported that the spiral waves could be formed in an inhomogeneous excitable medium with small-world connections under an optimal fraction of random connections. Wu et al. [18] gave detailed discussion about potential mechanism for pattern selection in neuronal network. Perc [19] discussed the effect of noise on the pattern formation in the small-world network. Ma et al. [20] detected the stability and transition of spiral wave of network by changing the probability of long range connection. Qin et al. [21] reported that target wave and spiral wave could be induced with feedback forcing current on the membrane potential with different certain time-delays and gains. Roxin et al. investigated the self-sustained wave formation of coupled neurons connected with small-world type. Wang et al. [23] reported that the time delay enhance the coherence of spiral waves for Hodgkin-Huxley neuronal with noise. Interestingly, spirals distribution was found in disinhibited mammalian neocortex [25–27] for experimental evidences, similar to the target wave, these ordered waves can regulate the collective behaviors of network like a continuous pacemaker. In fact, the formation and development of spatial regularity could be associated with the self-organization and competition among complex spatiotemporal system. In dynamical view, the developed states of spatiotemporal system and network depend on the initial states, topology connection (regular connection or small-world connection type), and bifurcation parameters. Li et al. [26] reported that the target wave and spiral wave could be induced by a localized inhomogeneity on spatiotemporal chaotic state, suggested that mechanisms underlying the inhomogeneity sustained coherent wave patterns seem quite different for oscillatory and stationary inhomogeneities. For example, defects block [28] can induce formation of spirals in network, external forcing with diversity [29], inhomogeneity in medium [30], noise[31–34], electric field depolarization [35–38] can greatly change the dynamics of spiral wave and target waves in the spatiotemporal system. Gosak et al. [31] studied the spatial dynamics of excitable media under the subthreshold periodic pacemaker activity and internal noise. Hou et al [32] investigated the spiral wave induced by the temporal noise, the spatial disorder and spatiotemporal fluctuation on the formation of spiral waves are discussed in detail. Tang et al. [33] confirmed that optimal noise could be useful to induce spirals in neuronal network under coherence resonance. Chen et al.[37] investigated the breakup of spiral wave and consequent patterns under the strong polarized advective field, the results shown that the symmetry and chirality of the applied external field are important to form the new-formed patterns. Jiang et al.[38] investigated the emergence of target waves in a cyclic predator-prey model, the results shown that the pattern formation resulting from the interplay between local and global dynamics in systems governed by cyclically competing species. That is to say, pattern formation is often associated with the collective behaviors of coupled oscillators or nodes. For a review about collective behaviors of coupled neurons and pattern transition, please refer to Refs.[39,40].

In the case of collective behaviors of coupled oscillators and network, the local kinetics of node is often supposed under periodical rhythm or chaotic property. Indeed, oscillator with multi-scroll attractors could show more complex dynamical behavior and the collective behavior of these coupled attractors could become more interesting. In dynamical control, many effective schemes [41–46] are used to generate multi-scroll attractors and wings in low-dimensional chaotic oscillators by replacing the nonlinear term with appropriate piecewise-linear functions or jerk function. In a feasible way, a type of Sine function is used to replace the nonlinear term in the Chua circuit so that infinite scroll attractors could be induced [46], and extensive results are reproduced in PSpice [47]. In this paper, we will study the pattern formation of coupled Chua circuits with infinite scrolls attractors, which are generalized by replacing the nonlinear term in classic Chua circuit [46] with a type of Sine function, so infinite equilibrium points are generated. Furthermore, a method of gradient negative feedback (feedback coefficient with diversity) is presented to generate certain ordered patterns. The network is divided into two regions: local square central region(A_2_ = *n*×*n*) and the rest part of the network(A_1_), and then the different negative feedback coefficients are imposed on the two parts respectively. It is found that different kinds of ordered patterns (spiral wave, target wave and breakup pattern) could be developed under different gradient feedback coefficients and appropriate sizes of feedback region (A_2_).

## Models, Schemes and Results

### Multi-scrolls attractors of Chua circuit

The generalized Chua circuit [45, 46] with nonlinear function is described as follows

$$\left\{ \begin{array}{l} \dot{x}=\alpha (y-f(x))\\ \dot{y}=x-y+z\\ \dot{z}=-\beta y \end{array}\right.$$ (1)

**Fig 1:**
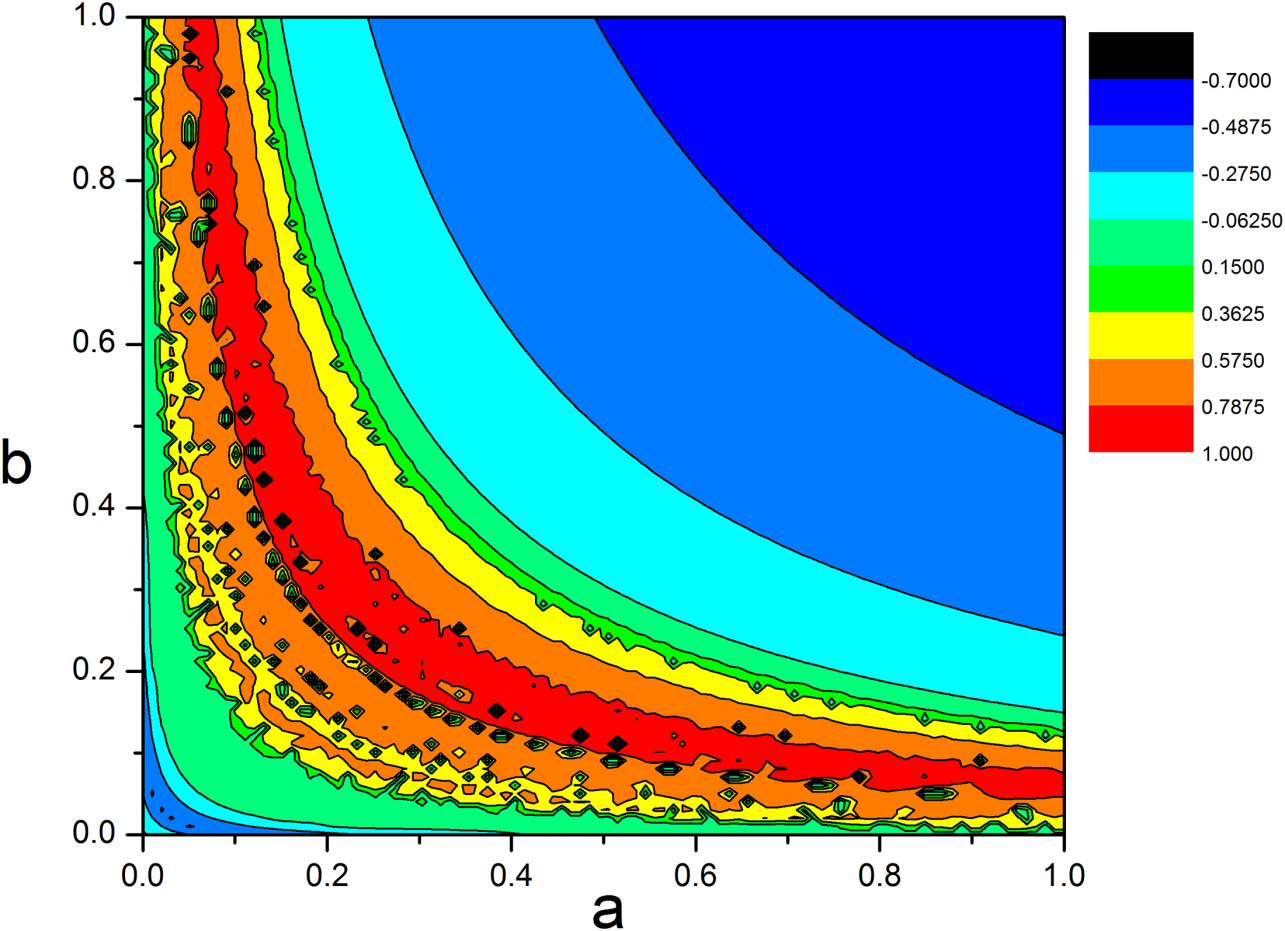
Distribution for the largest Lyapunov exponent for Eq 1 in the two-parameter space. The snapshots are plotted in color scale at *α* = 10.814, *β* = 14.

The parameter *α* = 10.814, *β* = 14, as reported in Ref.[45,46], the Josephson junction is effective to generate multi-scrolls attractor in the Jerk and Chua circuits driven by a Sine function. The underlying mechanism is considered as that the nonlinear system has a group of equilibrium points if the nonlinear term is described by a type of Sine function. As a result, we can replace the nonlinear term in Eq 1 with a similar Sine function, and it reads

f(x)=asin(2πbx) (2)

where *a*, *b* are parameters in Chua circuit, and then a group of equilibrium points are approached as (*n*/2*b*, 0, −*n*/2*b*), *n* = 0, 1, 2, 3,…. The largest Lyapunov exponent spectrum is calculated to find the parameter region for chaos emergence. As a result, the distribution in Fig 1 for the largest Lyapunov exponent spectrum of Eq 1 is plotted in the two-parameter space of *a* and *b*. The nonlinear system of Eq 1 is in chaotic state, when the largest Lyapunov exponent is positive.

**Fig 2:**
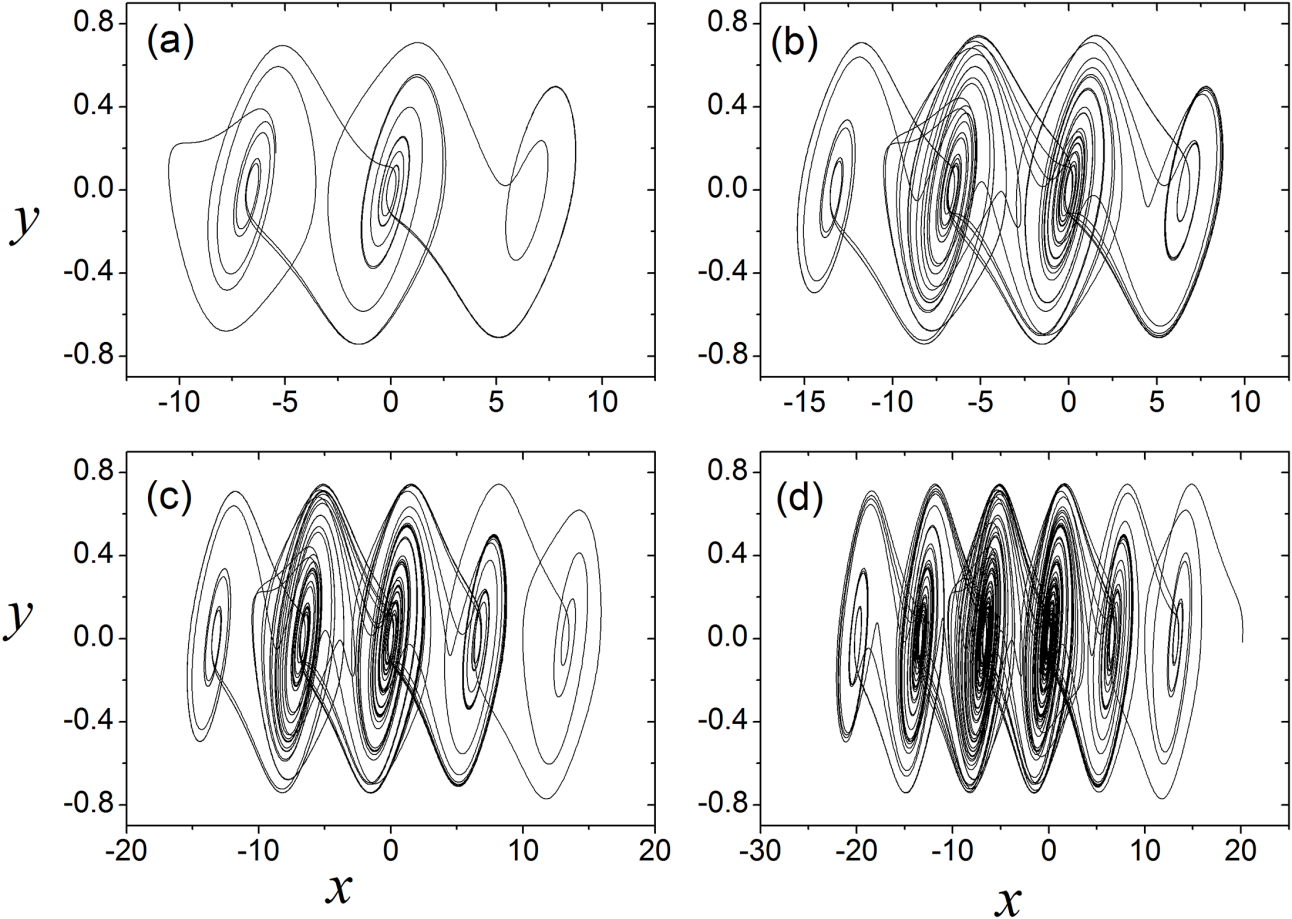
Multi-scrolls attractors are generated within different simulation time. For (**a)** *t* = 50 time units, for (**b)** = 200 time units, for (c) *t* = 300 time units, for (**d)** *t* = 500 time units, *α* = 10.814, *β* = 14, *a* = 0.2, *b* = 0.15.

It is found in Fig 1 that the appropriate parameter could induce the chaotic state in Eq 1. Furthermore, the phase portraits are shown in Fig 2 to discern the portrait of multi-scroll attractors. It is confirmed that the number of scrolls increases with calculating time because more equilibriums are generated driven by Sine function. Extensive results confirmed that Hamilton energy is decreased with increasing the number of attractors [48] in chaotic oscillator.

The results in Fig 2 indicated that the number of scrolls depends on the calculating time, and longer transient period is helpful to generate more scroll attractors in this chaotic oscillator. In fact, the collective behaviors of coupled multi-scroll attractors could become more complex. For simplicity, 200×200 Chua circuits with infinite-scrolls attractor are distributed in a two- dimensional lattice network with no-flux boundary condition being considered.

### The network of Chua circuits with infinite-scrolls attractor

**Fig 3:**
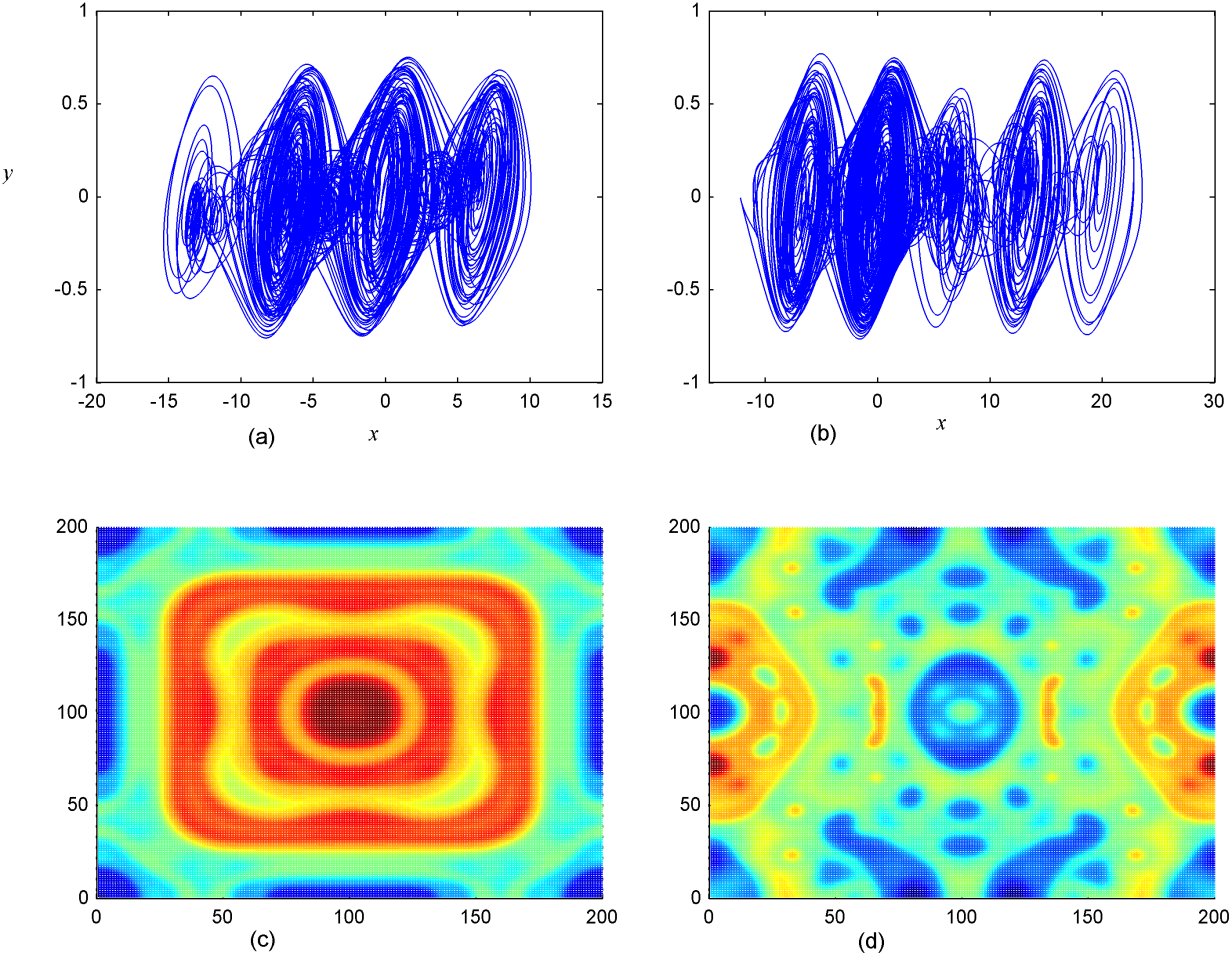
Attractors and spatial patterns. **(a-b)** the 4-scroll attractors for node (5,5) and 5-scroll attractor for node(50,50), respectively; The developed spatial pattern, for *t* = 40 **(c)**, *t* = 1000 **(d)** time units. The coupling intensity is selected as *k*_1_ = *k*_2_ = *k*_3_ = 5.5.

The network of the coupled Chua circuits with infinite-scrolls attractor with nearest-neighbor coupling is described by

$$\left\{ \begin{array}{l} {\dot{x}}_{ij}=\alpha (y_{ij}-a\text{sin}(2\pi bx_{ij}))+k_{\text{1}}(x_{ij+1}+x_{ij-1}+x_{i+1j}+x_{i-1j}-4x_{ij})\\ {\dot{y}}_{ij}=x_{ij}-y_{ij}+z_{ij}+k_{\text{2}}(y_{ij+1}+y_{ij-1}+y_{i+1j}+y_{i-1j}-4y_{ij})\\ {\dot{z}}_{ij}=-\beta y_{ij}+k_{\text{3}}(z_{ij+1}+z_{ij-1}+z_{i+1j}+z_{i-1j}-4z_{ij}) \end{array}\right.$$ (3)

where *α* = 10.814, *β* = 14, *a* = 0.2, *b* = 0.15, parameter *k*_1_, *k*_2_, *k*_3_ is couple intensity, and all the Chua circuits in the network are identical. Preliminary numerical simulation found that regular pattern can’t be formed with single channel coupling (*k*_1_≠0, *k*_2_ = 0, *k*_3_ = 0) and two-channel coupling (*k*_1_≠0, *k*_2_≠0, *k*_3_ = 0), but three-channel coupling (*k*_1_≠0, *k*_2_≠0, *k*_3_≠0) is helpful to induce spatiotemporal patterns. That is to say, one-channel or two-channel coupling can’t support regular spatial patterns but induce broken segments or spatiotemporal chaos in the network. To investigate the transition of spatial patterns, three-channel coupling is applied to induce regular patterns in the network. However, the developed patterns induced by three-channel coupling is not stable, it is changed with increasing time, and stable ordered patterns could not be induced with special initial values, the results are plotted in Fig 3.

The results in Fig 3 confirmed that the developed spatial patterns keep unstable and multi-stability states coexist with the increasing of number of scroll-attractors. Therefore, it is important to find feasible ways to stabilize these unstable patterns. Some general methods are checked to produce ordered pattern in the network described by Eq 3, such as: (i)Special initial values, (ii)Inhomogeneity medium, (iii)Periodic external forcing signal on the border or central region of the networks, (iv) Phase space compression. Unfortunately, extensive numerical results show that the stable ordered pattern can’t be developed under these conditions. Fortunately, gradient negative feedback is presented to induce the ordered pattern, and the results found the emergence of spiral wave, target wave and breakup pattern under appropriate conditions.

### Gradient Negative Feedback for the network

**Fig 4:**
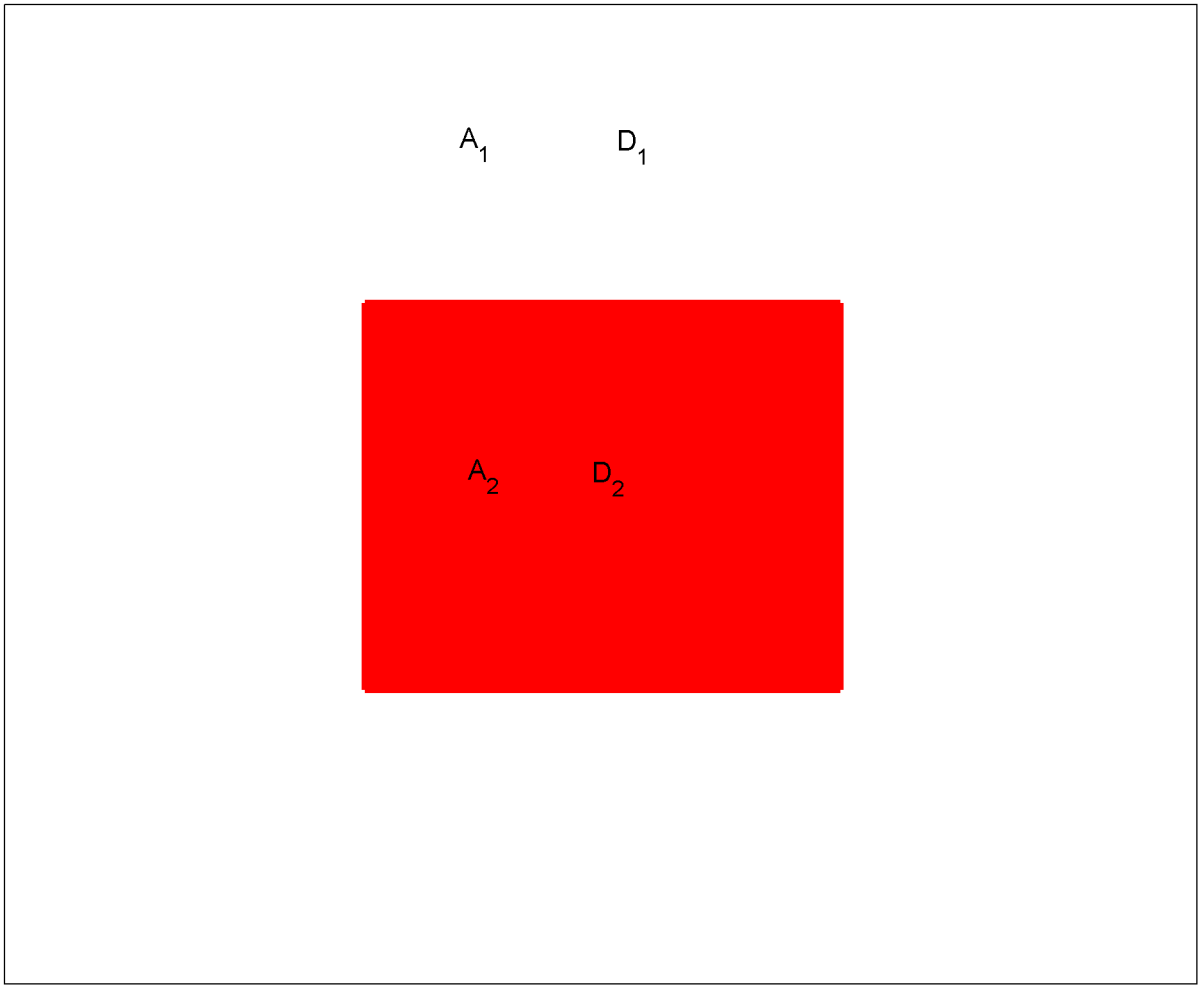
The schematic diagram for gradient negative feedback for network composed of 200×200 identical Chua circuits with infinite-scrolls attractor. *A*_2_ is the size of central square area (red area), and *D*_2_ is the feedback coefficient in the red *A*_2_ area; *A*_1_ is size of the rest area of network (white area), and *D*_1_ is the feedback coefficient in the white *A*_1_ area.

The method of gradient negative feedback is realized by dividing the network into two parts: central square area(*A*_2_ = *n*×*n*) (6×6, 11×11, 16×16, …,) and outer area of the network(*A*_1_), and then different values of negative coupling coefficients(*D*_1_ and *D*_2_) are imposed on the two areas(*A*_1_ and *A*_2_) of the network, respectively. For showing the method clearly, the schematic diagram is shown in Fig 4.

For simplicity, makes *k*_1_ = *k*_2_ = *k*_3_ = *k*, and the network driven by gradient feedback is described by

$$\left\{ \begin{array}{l} {\dot{x}}_{ij}=\alpha (y_{ij}-a\text{sin}(2\pi bx_{ij}))+k(x_{ij+1}+x_{ij-1}+x_{i+1j}+x_{i-1j}-4x_{ij})-Dx_{ij}\;\\ {\dot{y}}_{ij}=x_{ij}-y_{ij}+z_{ij}+k(y_{ij+1}+y_{ij-1}+y_{i+1j}+y_{i-1j}-4y_{ij})-Dy_{ij}\;\\ {\dot{z}}_{ij}=-\beta y_{ij}+k(z_{ij+1}+z_{ij-1}+z_{i+1j}+z_{i-1j}-4z_{ij})-Dz_{ij}\\ D=D_{1}\;for\;area\;A_{1},\;D=D_{2}\;for\;area\;A_{2} \end{array}\right.$$ (4)

**Fig 5:**
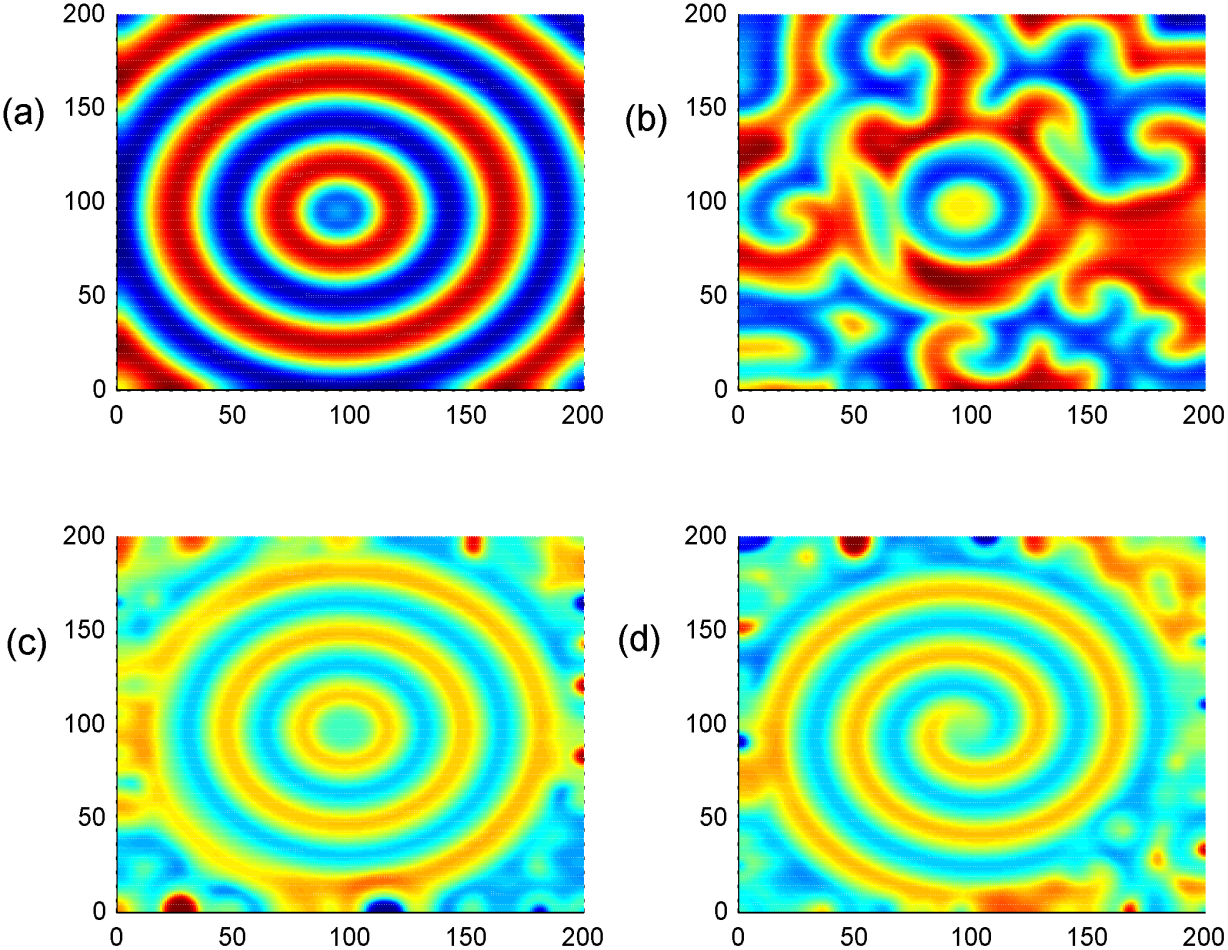
Formation of spatial patterns. **(a-d)** The developed spatial states with different conditions being used at *t* = 1000 time units; **(a)** stable target wave at *D*_1_ = 0.2, *D*_2_ = 0.5, *A*_2_ = 11×11(90≤*i*, *j*≤100); **(b)** broken patterns at *D*_1_ = 0.2, *D*_2_ = 0.5, *A*_2_ = 15×15; **(c)** stable target wave at *D*_1_ = 0.1, *D*_2_ = 0.4, A_2_ = 15×15; **(d)** spiral wave at *D*_1_ = 0.2, *D*_2_ = 0.5, *A*_2_ = 16×16 (90≤*i*, *j*≤105).

As a result, negative feedback is generated on the network by selecting negative feedback coefficients, *D*_1_ and *D*_2_. Indeed, the effect of negative feedback scheme mainly depends on three effective parameters *D*_1_, *D*_2_ and *A*_2_. In Eq 4, parameters *α* = 10.814, *β* = 14, *a* = 0.2, *b* = 0.15, *k* = 5.5, 200 ×200 oscillators are used in the two-dimensional array. It is found that spiral wave, target wave and breakup of patterns cam be observed under appropriate selection for parameters *A*_2_, *D*_2_, *D*_1_, and some results are shown in Fig 5.

The results in Fig 5 confirmed that the developed pattern selection much depends on the parameters in diversity as D_1_, D_2_, and A_2_.

As a result, it is interesting to explore the potential mechanism for this kind of wave formation by checking the time series and dynamical properties of the controlled area, and detailed numerical results could refer to Figs 6 to 15.

**Fig 6:**
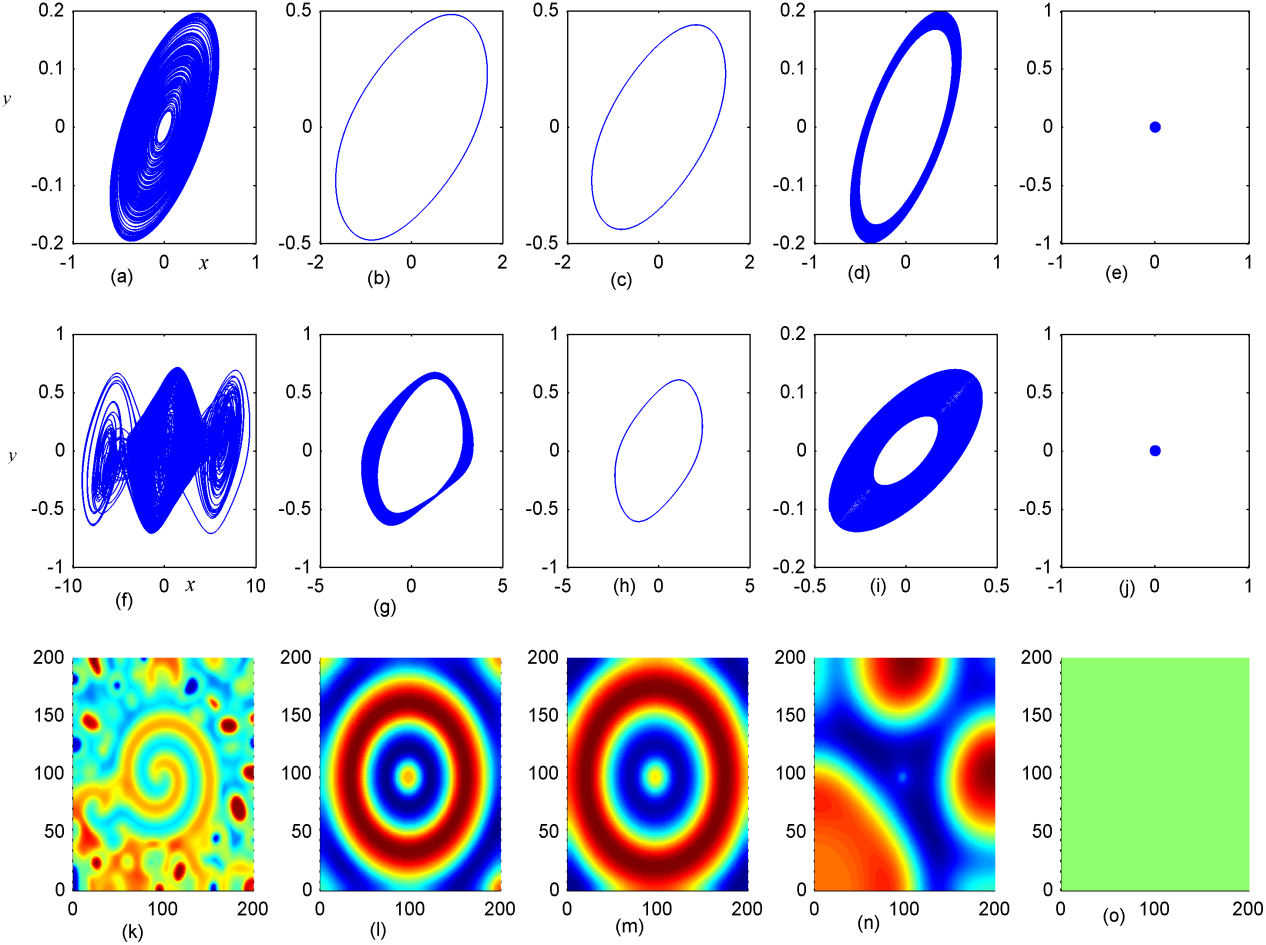
Stabilization of attractors and patterns driven by negative feedback. *A*_2_ = 6×6(*A*_2_ = 95≤*i*, *j*≤100), **(a-e)** the attractors for the node (98, 98) in *A*_2_ area; **(f-j)** the attractors for node (5, 5) in *A*_1_ area; **(k-o)** the snapshots for pattern of the network. **(a, f, k)** *D*_1_ = 0.1, *D*_2_ = 0.5, *t* = 4000 time units; **(b, g, l)** *D*_1_ = 0.2, *D*_2_ = 0.5, *t* = 1000; **(c, h, m)** *D*_1_ = 0.3, *D*_2_ = 0.5, *t* = 1000 time units; **(d, i, n)** *D*_1_ = 0.4, *D*_2_ = 0.5, *t* = 1000; **(e, j, o)** *D*_1_ = 0.5, *D*_2_ = 0.6, *t* = 1000 time units.

**Fig 7:**
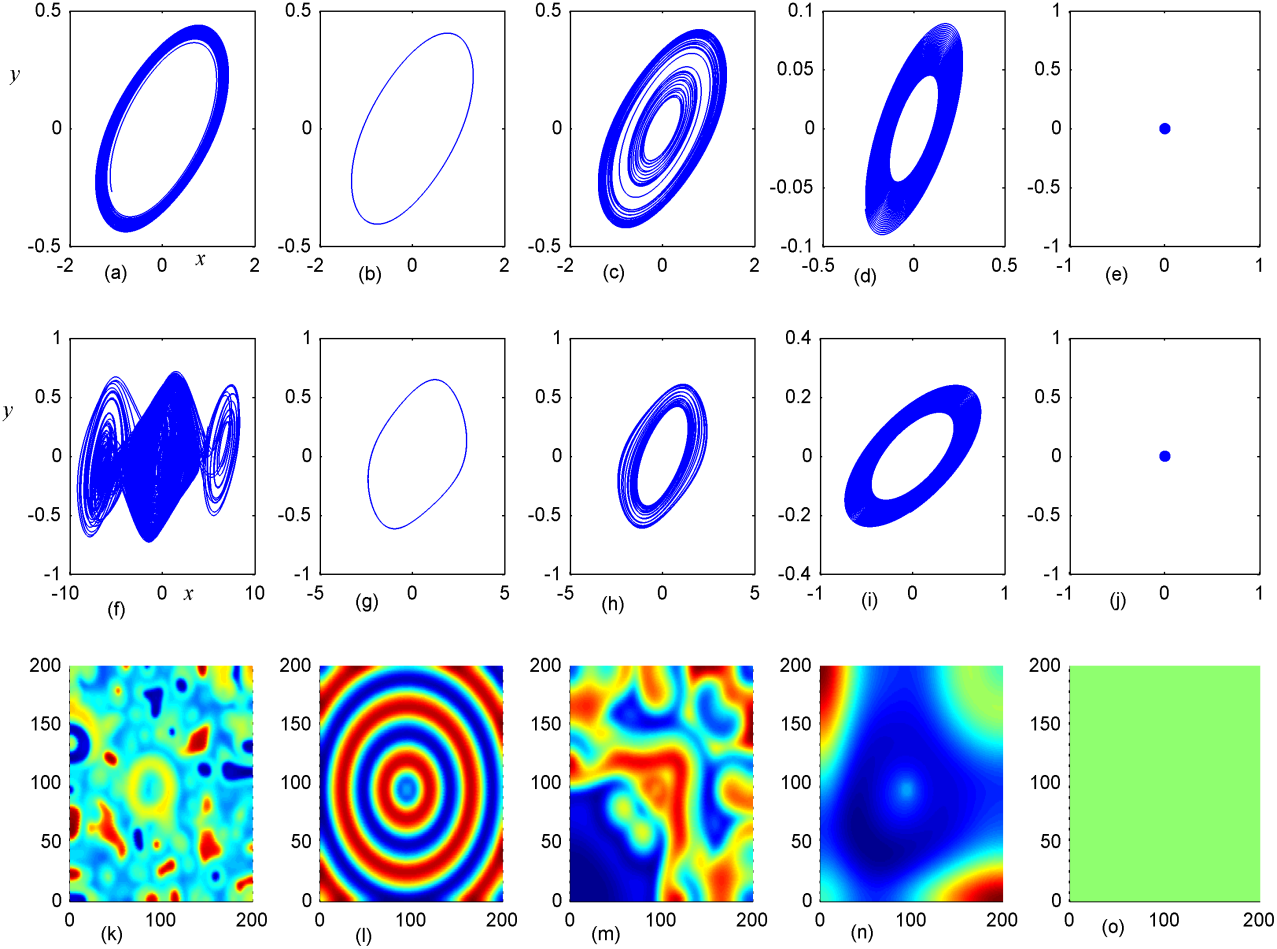
Stabilization of attractors and patterns driven by negative feedback. *A*_2_ = 11×11(*A*_2_ = 90≤*i*, *j*≤100), **(a-e)** the attractors for the node (98,98) in *A*_2_ area; **(f-j)** the attractors for node(5,5) in *A*_1_ area; **(k-o)** the snapshot for pattern **(a, f, k)***D*_1_ = 0.1, *D*_2_ = 0.5, *t* = 4000; **(b, g, l)** *D*_1_ = 0.2, *D*_2_ = 0.5, *t* = 1000;**(c, h, m)** *D*_1_ = 0.3, *D*_2_ = 0.5, *t* = 1000;**(d, i, n)** *D*_1_ = 0.4, *D*_2_ = 0.5, *t* = 1000; **(e, j, o)** *D*_1_ = 0.5, *D*_2_ = 0.6, *t* = 1000 time units.

**Fig 8:**
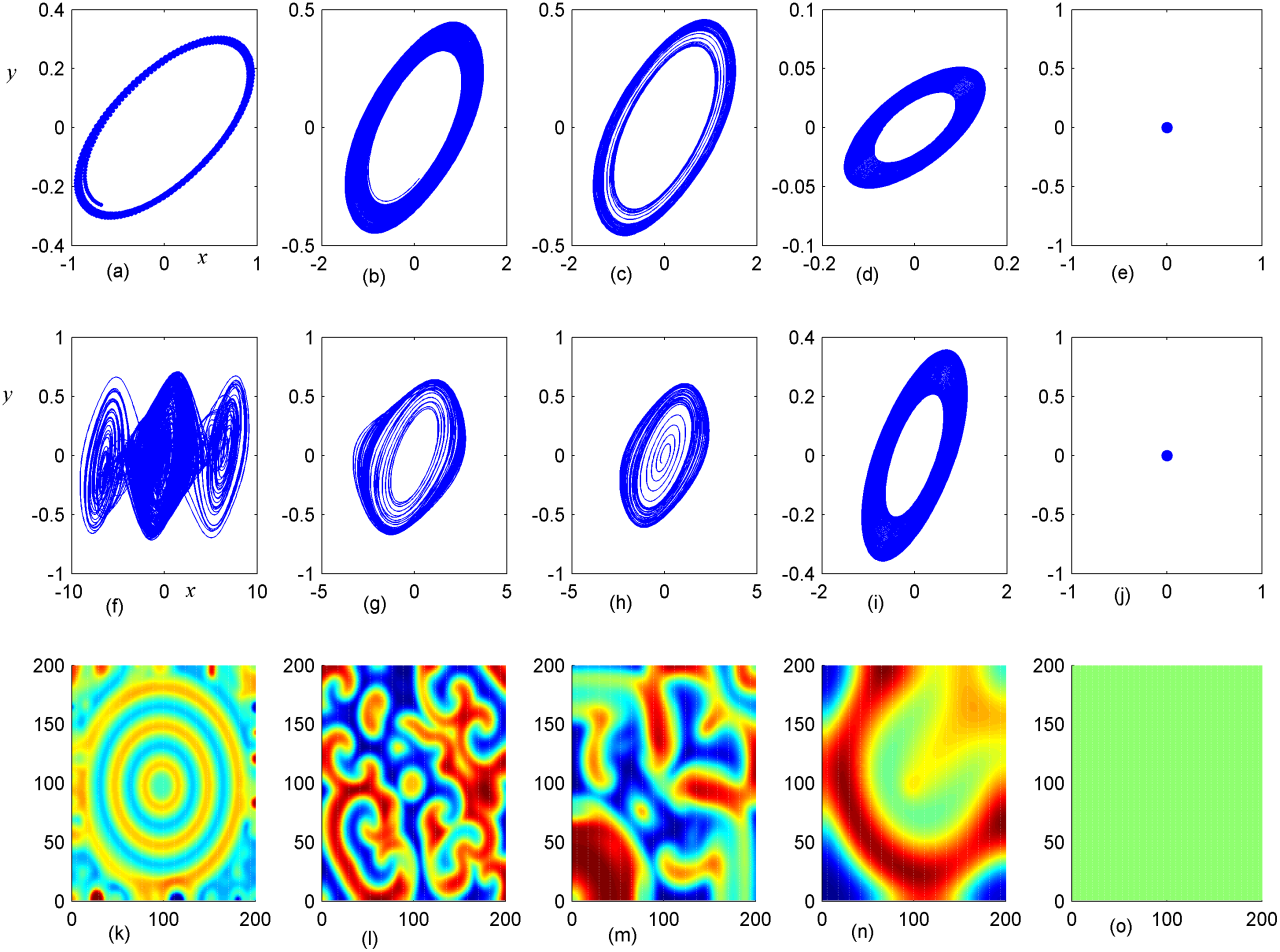
Stabilization of attractors and patterns driven by negative feedback. *A*_2_ = 16×16(A_2_ = 90≤*i*, *j*≤105), **(a-e)** the attractors for the node (98, 98) in *A*_2_ area; (**f-j)** the attractors for node (5,5) in *A*_1_ area; **(k-o)** the snapshot for pattern of the network. **(a, f, k)** *D*_1_ = 0.1, *D*_2_ = 0.4, *t* = 1000; **(b, g, l)** *D*_1_ = 0.2, *D*_2_ = 0.4, *t* = 1000; **(c, h, m)** *D*_1_ = 0.3, *D*_2_ = 0.4, *t* = 1000; **(d, i, n)** *D*_1_ = 0.4, *D*_2_ = 0.6, *t* = 1000; **(e, j, o)** *D*_1_ = 0.5, *D*_2_ = 0.6, *t* = 1000 time units.

**Fig 9:**
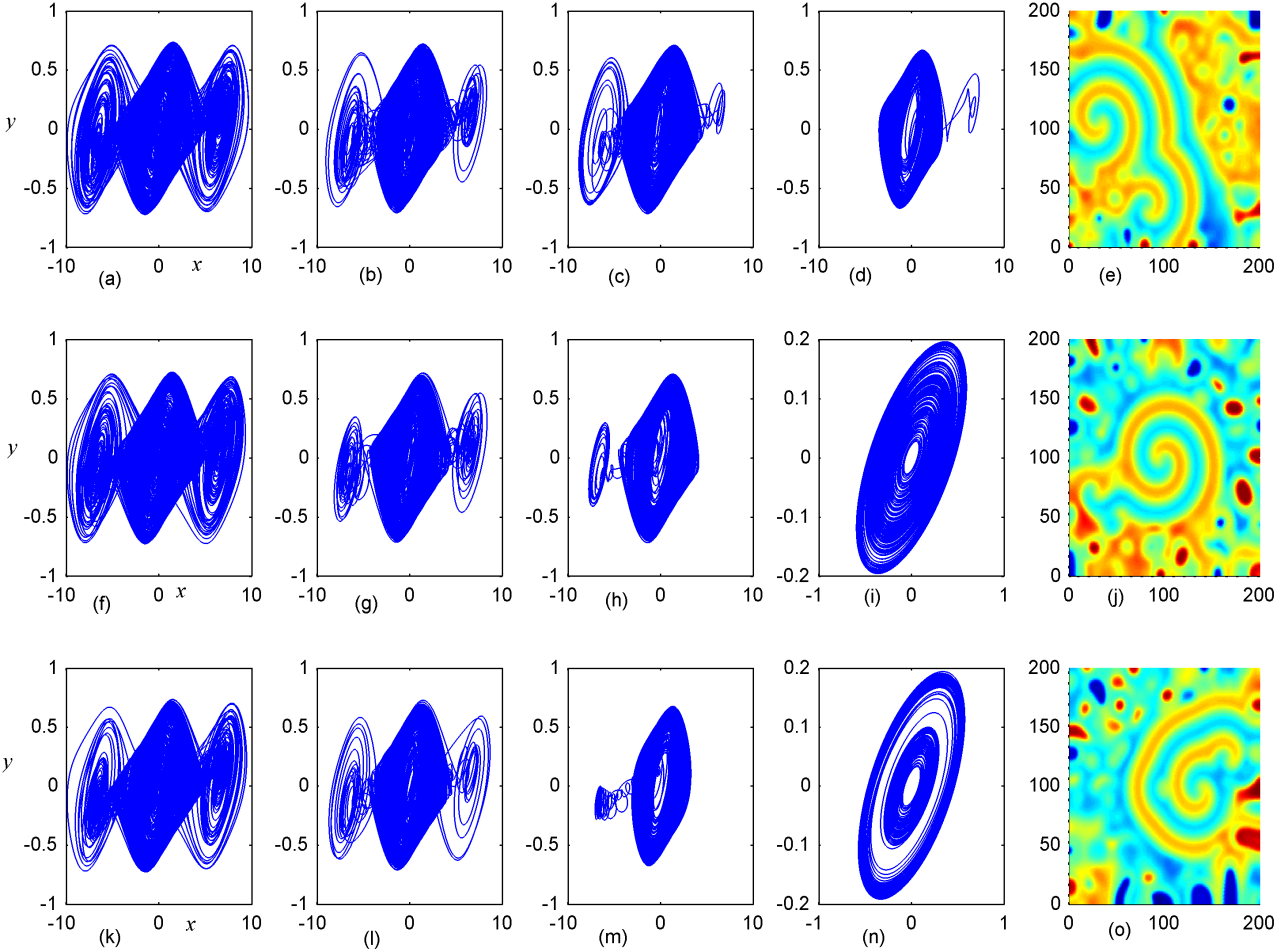
Stabilization of attractors and patterns driven by negative feedback. *D*_1_ = 0.1, *A*_2_ = 6×6(95≤*i*, *j*≤100), **(a-d)**, **(f-i)**, **(k-n)** the attractors for nodes(5,5), (10,10), (92,92), (98,98) respectively; **(e)**, **(j)**,**(o)** the snapshot for the developed states, respectively; **(a-e)** *D*_2_ = 0.2, *t* = 4000; **(f-j)** *D*_2_ = 0.5, *t* = 4000; **(k-o)** *D*_2_ = 2.0, *t* = 4000 time units.

**Fig 10:**
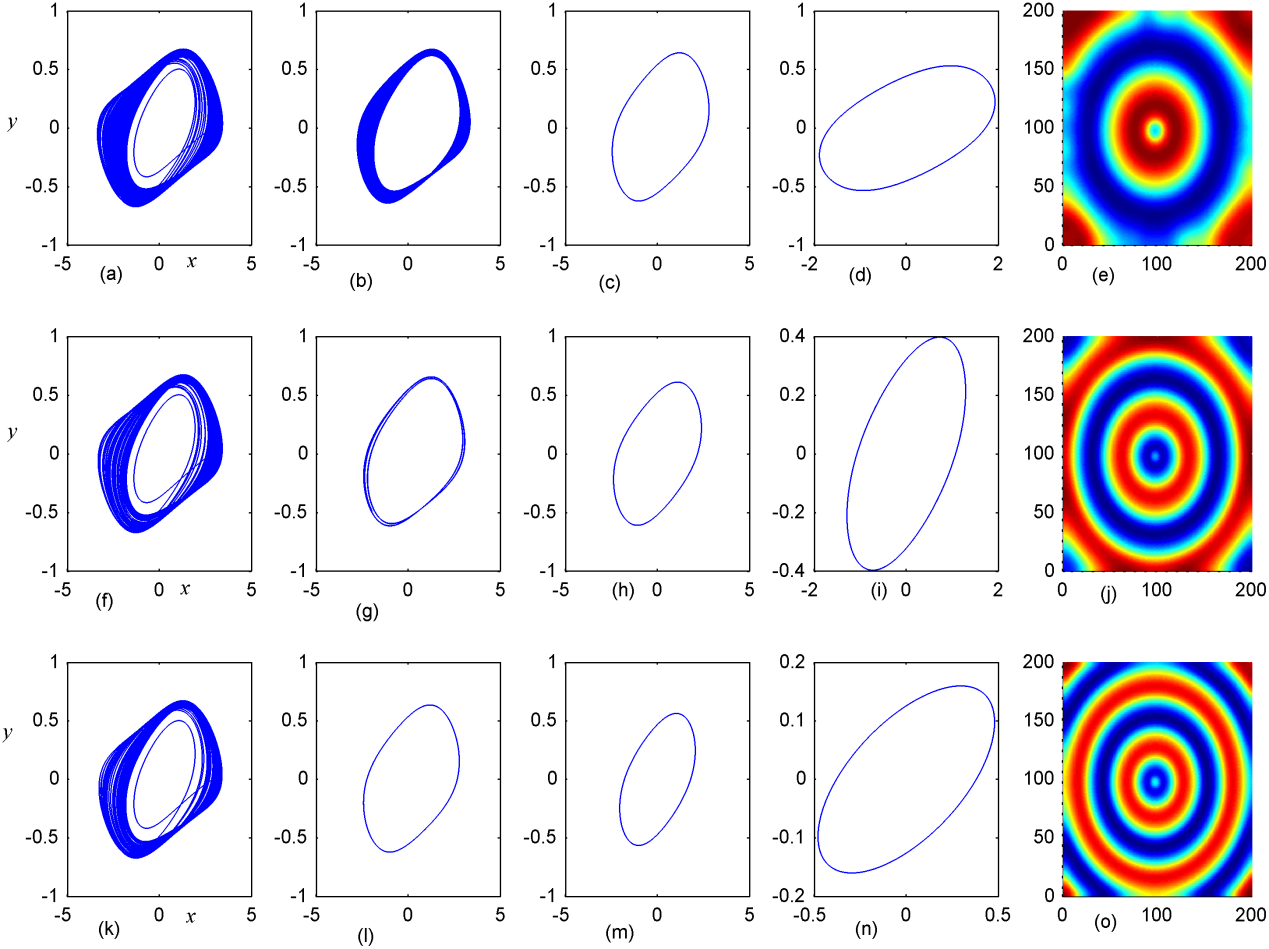
Stabilization of attractors and patterns driven by negative feedback. *D*_1_ = 0.2, A_2_ = 6×6(95≤*i*, *j*≤100), **(a-d),(f-i)**,**(k-n)** the attractors for nodes(5,5), (10,10), (92,92), (98,98) respectively; **(e)**,**(j)**,**(o)** the snapshot for the developed states, respectively; **(a-e)** *D*_2_ = 0.3, *t* = 1000; **(f-j)** D_2_ = 0.5, *t* = 1000; **(k-o)** D_2_ = 2.0, *t* = 1000 time units.

**Fig 11:**
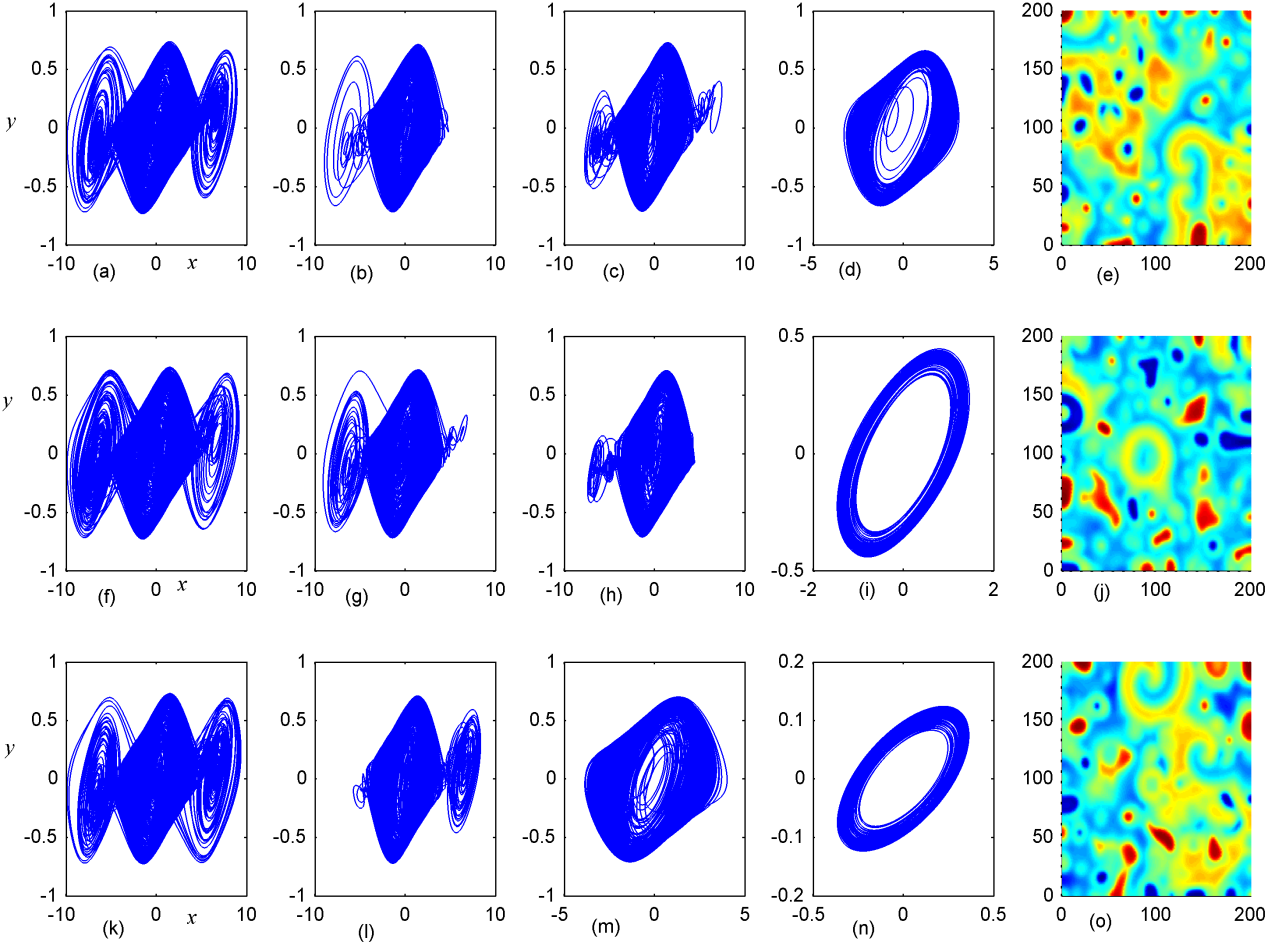
Stabilization of attractors and patterns driven by negative feedback. *D*_1_ = 0.1, *A*_2_ = 11×11(90≤*i*, *j*≤100), **(a-d),(f-i)**, **(k-n)** the attractors for nodes (5,5), (10,10), (85,85), (98,98), respectively; **(e)**,**(j)**,**(o)** the snapshot for the developed states, respectively; **(a-e)** *D*_2_ = 0.2, *t* = 4000; **(f-j)** *D*_2_ = 0.5, *t* = 4000; **(k-o)** *D*_2_ = 2.0, *t* = 4000 time units.

**Fig 12:**
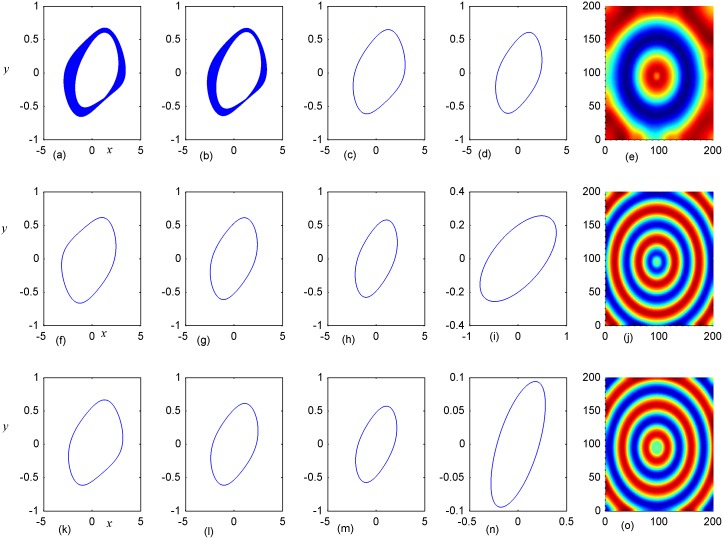
Stabilization of attractors and patterns driven by negative feedback. *D*_1_ = 0.2, *A*_2_ = 11×11(90≤*i*, *j*≤100), **(a-d),(f-i)**,**(k-n)** the attractors for nodes(5, 5), (10,10), (85, 85), (98, 98), respectively; **(e)**,**(j)**,**(o)** the snapshot for the developed states, respectively; **(a-e)** *D*_2_ = 0.3, *t* = 1000; **(f-j)** *D*_2_ = 0.8, *t* = 1000; **(k-o)** *D*_2_ = 2.0, *t* = 1000 time units.

**Fig 13:**
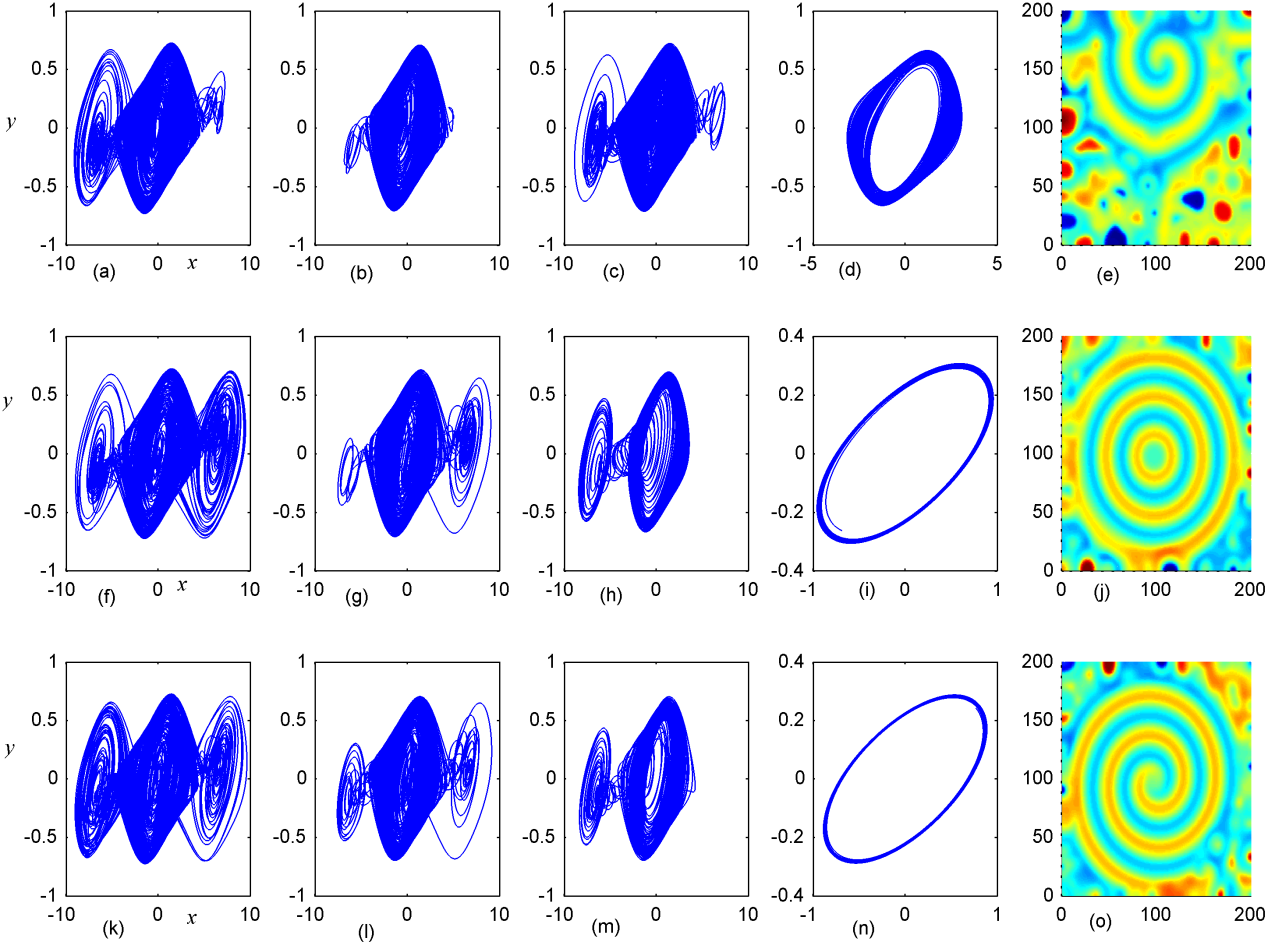
Stabilization of attractors and patterns driven by negative feedback. *D*_1_ = 0.1, *A*_2_ = 16×16 (90≤*i*,*j*≤105), **(a-d),(f-i)**,**(k-n)** the attractors for nodes(5,5), (10,10), (85,85), (98,98), respectively; **(e)**,**(j)**,**(o)** the snapshot for the developed states, respectively; **(a-e)** *D*_2_ = 0.2, *t* = 4000; **(f-j)** *D*_2_ = 0.4, *t* = 1000; **(k-o)** *D*_2_ = 0.5, *t* = 1000 time units.

**Fig 14:**
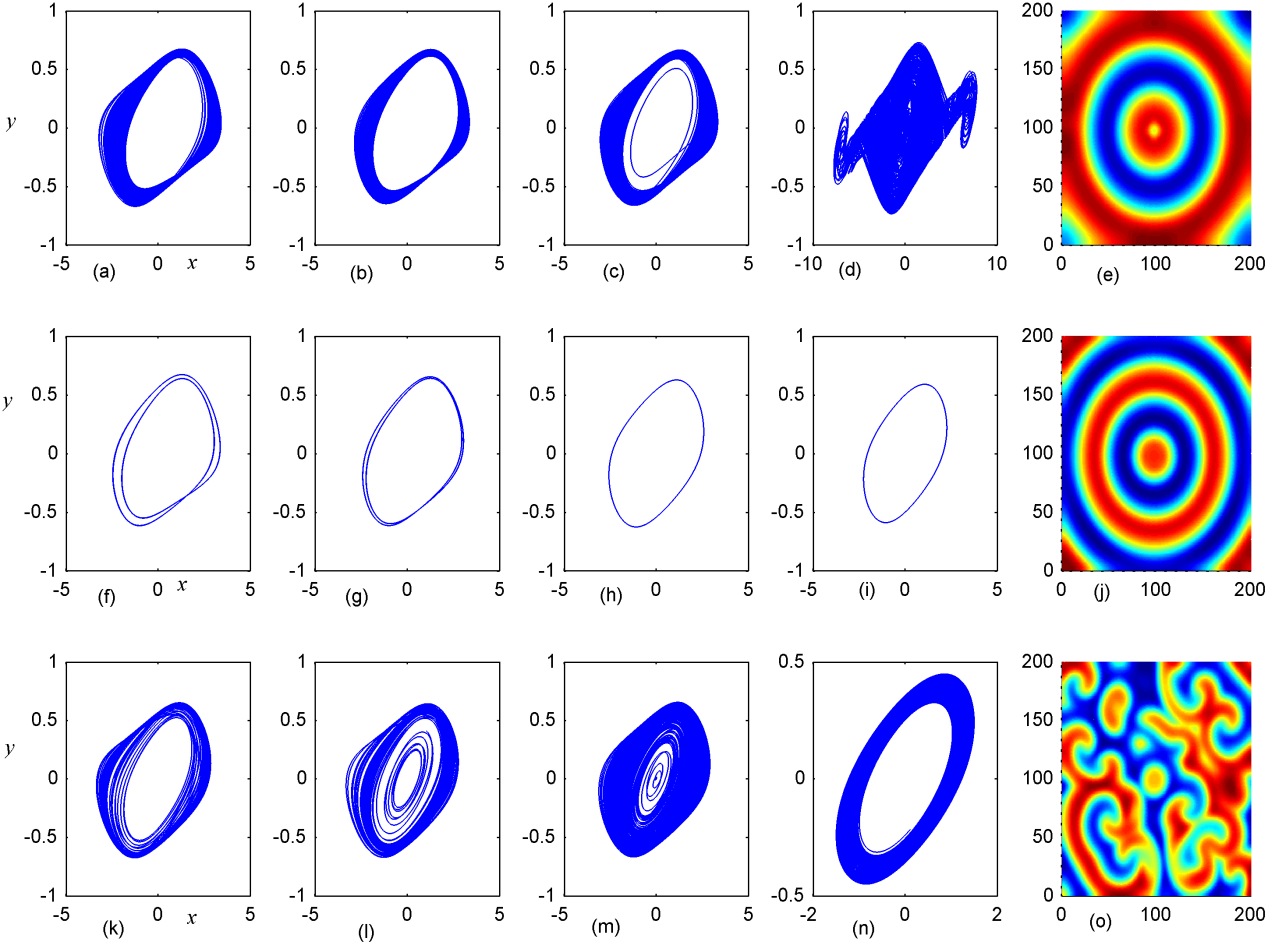
Stabilization of attractors and patterns driven by negative feedback. *D*_1_ = 0.2, *A*_2_ = 16×16(90≤*i*,*j*≤105), **(a-d), (f-i)**, **(k-n)** the attractors for nodes(5,5), (10,10), (85,85), (98,98), respectively; **(e)**,**(j)**,**(o)** the snapshot for the developed states, respectively; **(a-e)** *D*_2_ = 0.1, *t* = 1000; **(f-j)** *D*_2_ = 0.3, *t* = 1000; **(k-o)** D_2_ = 0.4, *t* = 1000 time units.

**Fig 15:**
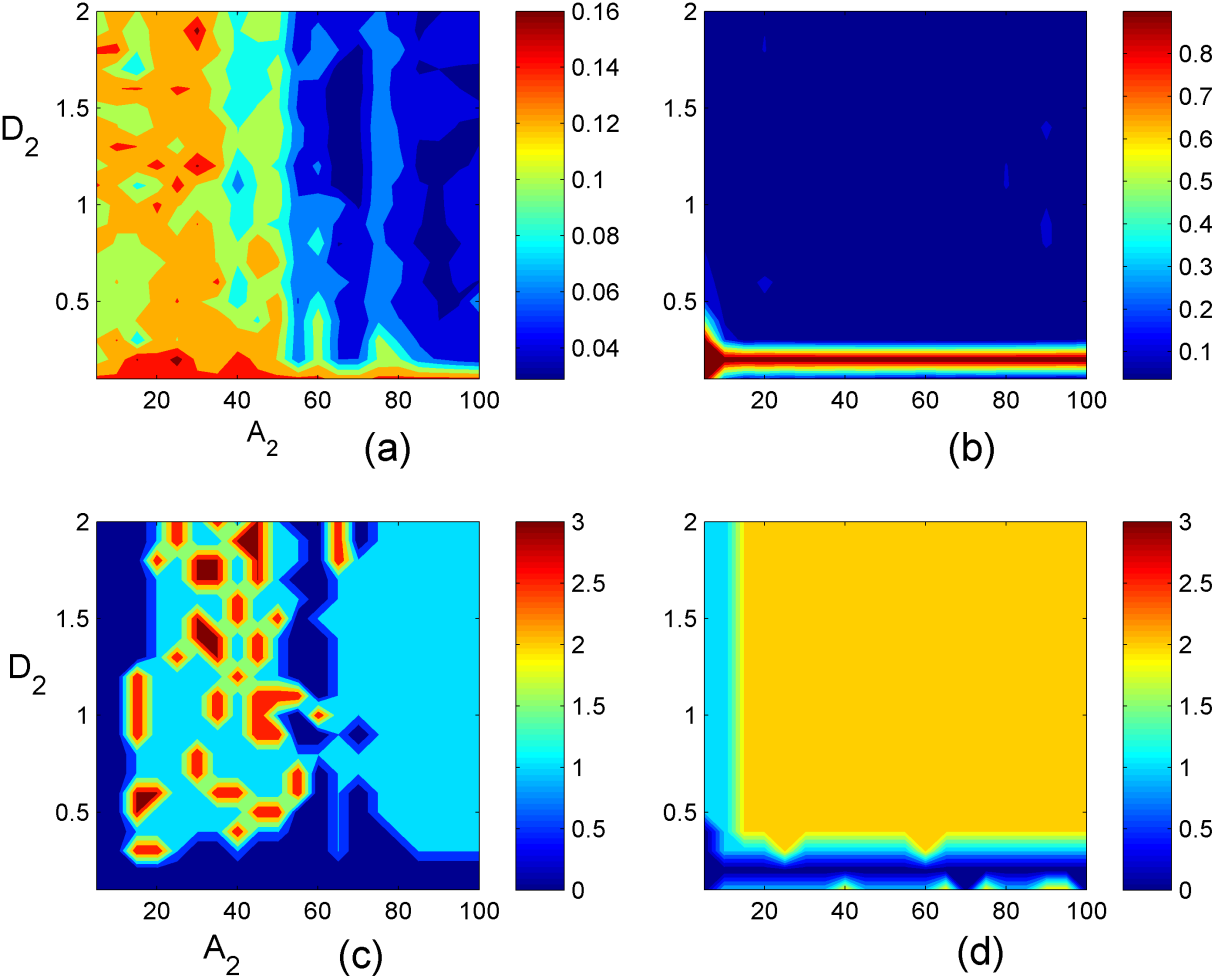
Distribution of factor of synchronization. **(a)** *D*_1_ = 0.1, the distribution of factor of synchronization *R* in the two-parameter space *A*_2_ and *D*_2_; **(b)** *D*_1_ = 0.2 the distribution of factor of synchronization R in the two-parameter space *A*_2_ and *D*_2_; **(c)** *D*_1_ = 0.1, the distribution of different patterns in the two-parameter space *A*_2_ and *D*_2_; **(d)** *D*_1_ = 0.2, the distribution of different patterns in the two-parameter space *A*_2_ and *D*_2_. (unstable pattern for 0-navy blue; target wave for 1-sky blue; broken patterns for 2-yellow; spiral wave for 3-red).

Firstly, the effect of factor *D*_1_ on the formation of ordered pattern is investigated. For simplicity, the value of *D*_1_ is selected from 0.1 to 0.5, and the values of D_2_, A_2_ are fixed certain values. Detailed results are calculated when A_2_ area is set as 6×6, 11×11, 16×16, respectively, and the results are plotted in Figs 6–8.

The results in Fig 6 indicated that spiral wave, target wave, homogeneous state could be developed in the network with increasing the value of *D*_1_ from 0.1 to 0.5. Compared the portraits in the first and second panel in Fig 6, it is found that regular spatial distribution could be supported and thus stable spatial patterns could be developed only when the periodicity of the sampled series from area *A*_2_ is approached. Homogeneous state is reached when the center area driven by negative feedback is decreased to stable state. The potential mechanism could be that negative feedback in the center area decrease the chaotic state to periodic or quasi-periodical state even stable state by fixing appropriate feedback coefficients. As a result, a continuous periodic driving emitted from the center areas as a pacemaker thus the collective behaviors of the network could be regulated; finally, stable spatiotemporal patterns could be developed completely. Furthermore, the size of *A*_2_ area is increased to 11×11 and 16×16, and the results are plotted in Figs 7 and 8.

It is found that dynamic properties of sampled series from the same node presented different behaviors when negative feedback on the center area is increased in size, and the collective behaviors and spatial patterns are changed greatly. Surely, negative feedback with stronger intensity just stabilizes the oscillators and the network thus homogeneous states are reached.

By further increasing the center area driven by negative feedback, it is found that stable target wave could be developed under smaller feedback gain in area *A*_1_ and breakup of spatial patterns are also induced by further increasing the feedback gain in center area *A*_1_. Indeed, the sampled series from nodes in area *A*_1_ keep irregular even chaotic and the collective disordered behaviors can’t be suppressed even the center control area is enlarged. The results in Figs 6, 7 and 8 confirmed that the developed state and dynamical behaviors of network just depends on the diversity between *D*_1_ and *D*_2_ thus gradient driving could be induced to emit continuous forcing like pacemaker, and the size of center controlled area A_2_ also played important role in changing the developed states of the network. In this way, further investigations were carried out thus the effect of feedback gain *D*_2_ and area size could be understood. For simplicity, the feedback gain *D*_2_ in center area *A*_2_ (6×6 nodes) is changed at fixed *D*_1_ = 0.1, 0.2, and the formation of spatial pattern in calculated in Figs 9 and 10. In Figs 11 and 12, different feedback gains *D*_2_ are used in the center control area *A*_2_ (11×11 nodes) by fixing the feedback gain *D*_1_ = 0.1, 0.2 in the outer area A_1_. Furthermore, the center area is increased to *A*_2_ (16×16 nodes) at fixed *D*_1_ = 0.1, 0.2 when different feedback gains *D*_2_ is used in the center control area *A*_2_, respectively, and the results are plotted in Figs 13 and 14.

It is confirmed in Fig 9 that spiral waves could be developed in the network for *D*_1_ = 0.1 that appropriate diversity in feedback gain is critical to induce continuous travelling waves, which encounter breakup close to the boundary between the two control area of network. Unfortunately, the developed spiral wave just covers certain area of the network and failed to grow up in the network completely. As a result, the phase portraits for the sampled oscillators or nodes often present chaotic attractor instead of periodical periodicity. That is to say, the coexistence between periodic attractors and multi-scroll attractors makes the spiral wave occupy the network in certain size instead of the full area completely.

By further increasing the feedback gain in area A_1_ that the diversity in feedback is decreased, it is found in Fig 10 that target wave could be developed because the gradient diversity can’t break the target waves emitted from the center area. Particularly, limit circle was observed from the sampled series of monitored nodes, and it indicated distinct periodicity is detected thus continuous target waves are developed in the network. Unfortunately, broken spatial patterns are formed by increasing the center control area *A*_2_ (11×11 nodes), and the results are calculated in Fig 11.

Similar to the results in Fig 9, spiral segments were observed in local area and most of the network was covered by broken segments, and the phase portraits for the sampled nodes presented multi-scroll or chaotic attractors. It indicated that diversity in feedback gain between the two control areas is not effective to induce stable travelling waves. Furthermore, the diversity between feedback gains from the two areas is decreased, and stable target waves could be developed in Fig 12.

The sampled series from the monitored nodes presented distinct periodicity and limit circles are detected when the center controlled area is increased in size under appropriate diversity in feedback gains, and stable target wave began to occupy the network completely. Furthermore, the center controlled area is increased and appropriate feedback gain with diversity is selected to generate perfect spiral wave so that the collective behaviors of network could be regulated by the self-sustained spiral waves, and the results are plotted in Fig 13.

It is interesting to find perfect spiral wave and/or target wave could be developed to occupy the network completely. It was confirmed that appropriate diversity in feedback gain and size of the controlled area are effective to support spiral wave and target wave as well.

Above all, most of cases have been investigated when the feedback gain in the center area A_2_ was selected with larger value than the outer area *A*_1_ (*D*_2_ >*D*_1_). To further verify the effect of diversity between feedback gain, it is interesting to investigate some cases under *D*_2_ <*D*_1_ that similar diversity in feedback gain could be generated, and some results are plotted in Figs 14 and 15 to understand the effect of feedback gain and control area in the formation of spatial patterns.

In the case of *D*_1_ = 0.1, the target wave or spiral wave could be induced by setting appropriate value for D_2_ and A_2_. However, for the case *D*_1_ = 0.2, the target wave is only stable with *A*_2_ ≤ 16×16 and *D*_2_ ≤0.3, otherwise, breakup of patterns occurs.

Finally, it is interesting to discern some statistical properties of the network during the formation of spatial patterns. Based on the mean filed theory, a statistical factor of synchronization [49] is redefined in the two parameter space [20, 21] to study the robustness of the pattern. The factors of synchronization *R* is calculated as follows

$$F=\frac{\text{1}}{N^{\text{2}}}\sum_{j=1}^{N}\sum_{i=1}^{N}x_{ij}=<x_{ij}{>}_{s}$$ (5)

$$R=\frac{\langle F^{2}\rangle -{\langle F\rangle }^{2}}{\frac{1}{N^{2}}\sum {}_{j=1}^{N}\sum {}_{i=1}^{N}(\langle x_{ij}^{2}\rangle -{\langle x_{ij}\rangle }^{2})}$$ (6)

The variable *F* is the spatial average value of the detected variables for each node in the network, <*> indicates the average for calculating time or period. N^2^ is the number of nodes in the network, the factor of synchronization *R* is calculated to detect the transition and stability of developed patterns. It is confirmed that a smaller value *R* is associated to an ordered state and non-perfect synchronization. Complete perfect synchronization is approached when the factor of synchronization R is much close to 1. As mentioned above, the developed states (spiral wave, target wave, homogeneous state) depend on the selection for feedback gains (*D*_1_, *D*_2_) and the size of center controlled areas A_2_. For simplicity, we calculated the distribution of factor of synchronization in the two-parameter space (*D*_2_ *vs*.*A*_2_) at fixed feedback gain *D*_1_ = 0.1 and *D*_1_ = 0.2, respectively. Furthermore, the pattern region is also calculated in the two-parameter space by detecting the developed states at *t* = 1000 time units. For simplicity, (1) if the pattern could not be induced within 1000 time unites, it is marked as number 0; (2) if target wave is formed within 1000 time unites, it is labeled as 1; if the pattern is breakup, be labeled as 2; (3) if spiral wave is developed, it is labeled as 3, these results are plotted in Fig 15.

According to the distribution region in Fig 15, it is confirmed that the developed states and pattern formation are greatly dependent on the selection of feedback gain and the size of the center controlled area as well. Appropriate selection for feedback gain with diversity and size of controlled area can generate appropriate spiral wave, target wave, homogeneous states in the network. The network or the media can show distinct periodicity when spiral wave and/or target wave is developed to occupy the network, and the sampled time series from monitored nodes can present distinct periodicity, and limit circle could also be observed in phase portrait. Surely, network of coupled periodic oscillators even chaotic oscillators can support the emergence of target wave and spiral wave, however, network composed of multi-scroll chaotic oscillators could be passive to support stable ordered waves because its complex dynamical properties in each node that multi-scroll attractors are associated with calculation time. Indeed, spatiotemporal chaos or broken segments in the network could be further suppressed by generating continuous spiral wave or target wave played as a powerful pacemaker.

As mentioned above, statistical factor of synchronization is effective to measure the phase transition and synchronization degree of the collective behaviors of network. Indeed, it is also important to study the synchronization stability of the isolate circuit or node of the network by using the master function approach [50, 51], and the basin area is calculated to discern the dependence of states on selection of initial values, furthermore, bifurcation analysis is also supplied.

### The stability of synchronization of the system under multi-variables (channels) coupling

**Fig 16:**
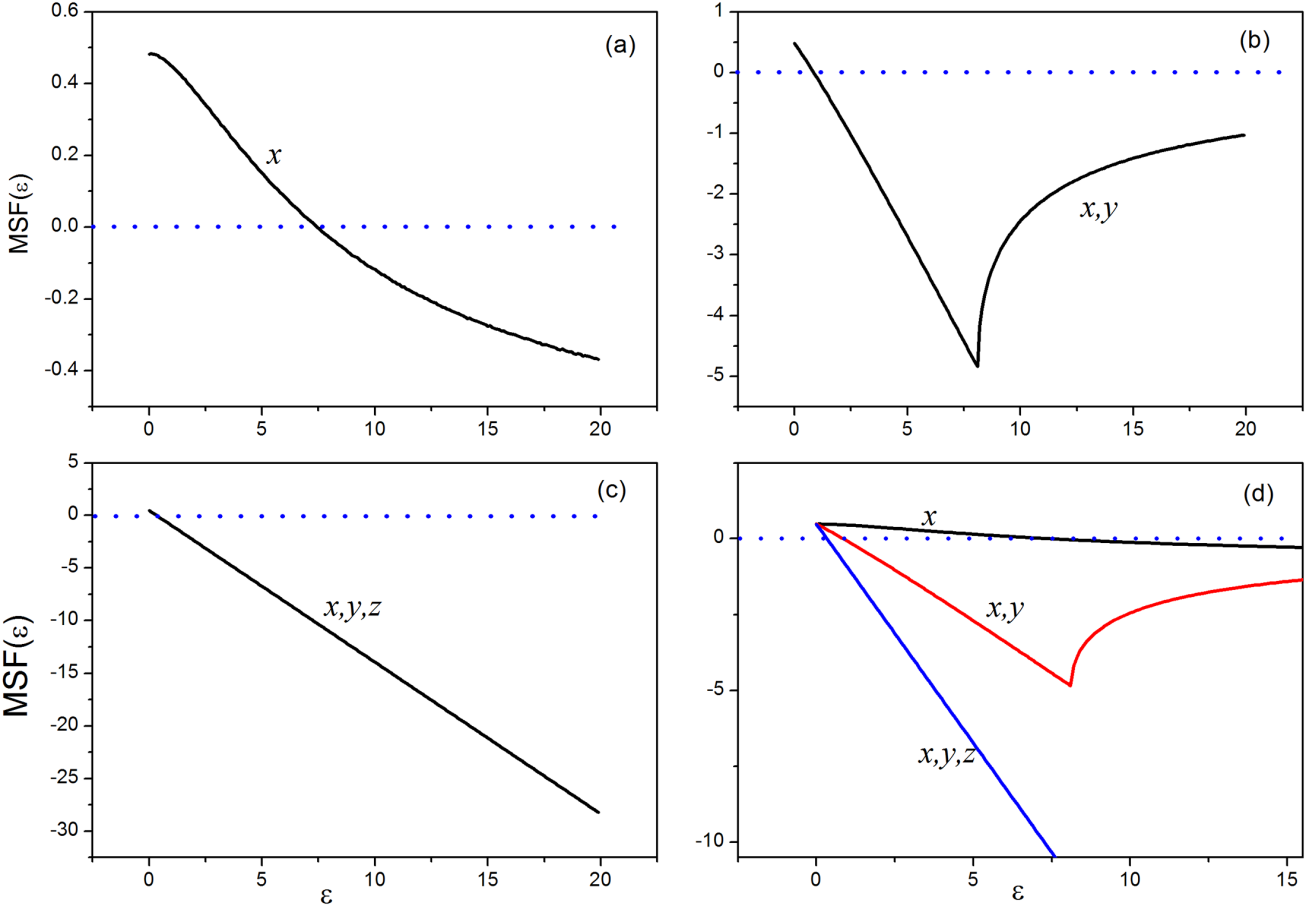
The master stability function MSF(ɛ) as a function of parameter ɛ. **(a)** *x* variable couple, **(b)** two variables coupling (*x*,*y*), **(c)**three variables coupling(*x*,*y*,*z*), **(d)** the results for three kinds of cases are plotted in one figure.

The previous works confirmed that spatial patterns could be selected due to three-channels coupling. To clarify this problem, the dependence of stability of the synchronization on the number of variables coupling is studied by the master stability function. The Eq 3 are written as follows

$$\left\{ \begin{array}{l} {\dot{x}}_{i}=\alpha (y_{i}-f(x_{i}))+k_{1}\sum_{j=1}^{N}A_{ij}(x_{j}-x_{i})\\ {\dot{y}}_{i}=x_{i}-y_{i}+z_{i}+k_{2}\sum_{j=1}^{N}A_{ij}(y_{j}-y_{i})\\ {\dot{z}}_{i}=-\beta y_{i}+k_{3}\sum_{j=1}^{N}A_{ij}(z_{j}-z_{i}) \end{array}\right.$$ (7)

where matrix *A*_*ij*_ is coupling matrix, according to the master stability function formalism, the Eq 7 are described by

$$\begin{array}{l} {\vec{w}}_{i}=F({\vec{w}}_{i})-k_{1}\sum_{i=1}^{N}L^{x}{}_{ij}H(x_{i})-k_{2}\sum_{i=1}^{N}L^{y}{}_{ij}H(y_{i})-k_{3}\sum_{i=1}^{N}L^{z}{}_{ij}H(z_{i})\\ \;=F({\vec{w}}_{i})-k\sum_{i=1}^{N}L_{ij}H({\vec{w}}_{i})\; \end{array}$$ (8)

where $F(\vec{w})$ is dynamical function for the system, Laplacian matrix is selected as the same $L_{ij}^{x},L_{ij}^{y},L_{ij}^{z}=L_{ij}$. H(*x*), H(*y*), H(*z*), H(***w***) are coupling functions. According to the master stability function presented by Pecora and Carroll [50, 51], the Lyapunov exponents can be determined from a single function that is independent of the network. Therefore, the master stability function for the Eq 8 could be written

$$\delta \dot{w}=(DF-\varepsilon DH)\delta w$$ (9)

$$DF=\left(\begin{array}{lll} -\alpha 2\pi ab\text{cos}(2\pi bx_{0}) & \;\alpha \; & 0\\ 1 & -1 & 1\\ 0 & -\beta & 0 \end{array}\right)$$ (10)

where *DF* and *DH* are the Jacobian matrices of functions F and H respectively, and *ε* = *kλ*_*k*_, *λ*_*k*_ is the eigenvalue of the Laplacian matrix, *x*_0_ is the equilibrium point, and *x*_0_ = *n*/(2*b*). As reported in Ref [52], the stability of the synchronization of the system could be affected by the multi-variables coupling. For simplicity, we will analyze the stability of synchronization of the system with only one, two or three variables coupling by Master stability function, respectively. Here the Jacobian matrices for coupling function *H*(***w***) with different numbers of variables coupling is described by.

$$DH_{x}=\varepsilon \left(\begin{array}{ccc} 1 & \;0 & 0\\ 0 & \;0 & 0\\ 0 & 0 & 0 \end{array}\right);DH_{x,y}=\varepsilon \left(\begin{array}{ccc} 1 & \;0 & 0\\ 0 & \;1 & 0\\ 0 & \;0 & 0 \end{array}\right);DH_{x,y,z}=\varepsilon \left(\begin{array}{ccc} 1 & \;0 & 0\\ 0 & \;1 & 0\\ 0 & \;0 & 1 \end{array}\right)$$ (11)

where *DH*_*x*_, *DH*_*x*,*y*_, *DH*_*x*,*y*,*z*_ is the Jacobian matrix for *x* variable, two variables(*x*,*y*) and there variables coupling(*x*,*y*,*z*), respectively. The maximum Lyapunov exponents of Eq 9 as a function of ε are calculated with the three kinds of coupling, respectively. The results are shown in Fig 16.

The results in Fig 16 indicate that the synchronization stability is enhanced with increasing the coupling channels or variables. The stability of synchronizability of the system under three- variables coupling is better than case for two-variables coupling, and two-variables coupling is better than single-variable coupling. Therefore, the synchronizability of the system could be enhanced by multi-variables coupling. As a result, we mainly discussed the pattern selection and control when nodes are coupled under three-channels. Indeed, the coupling between nodes imposed possible feedback on each node, and it is interesting to the stability of node or circuit under negative feedback.

### The analysis of isolated system with negative feedback

**Fig 17:**
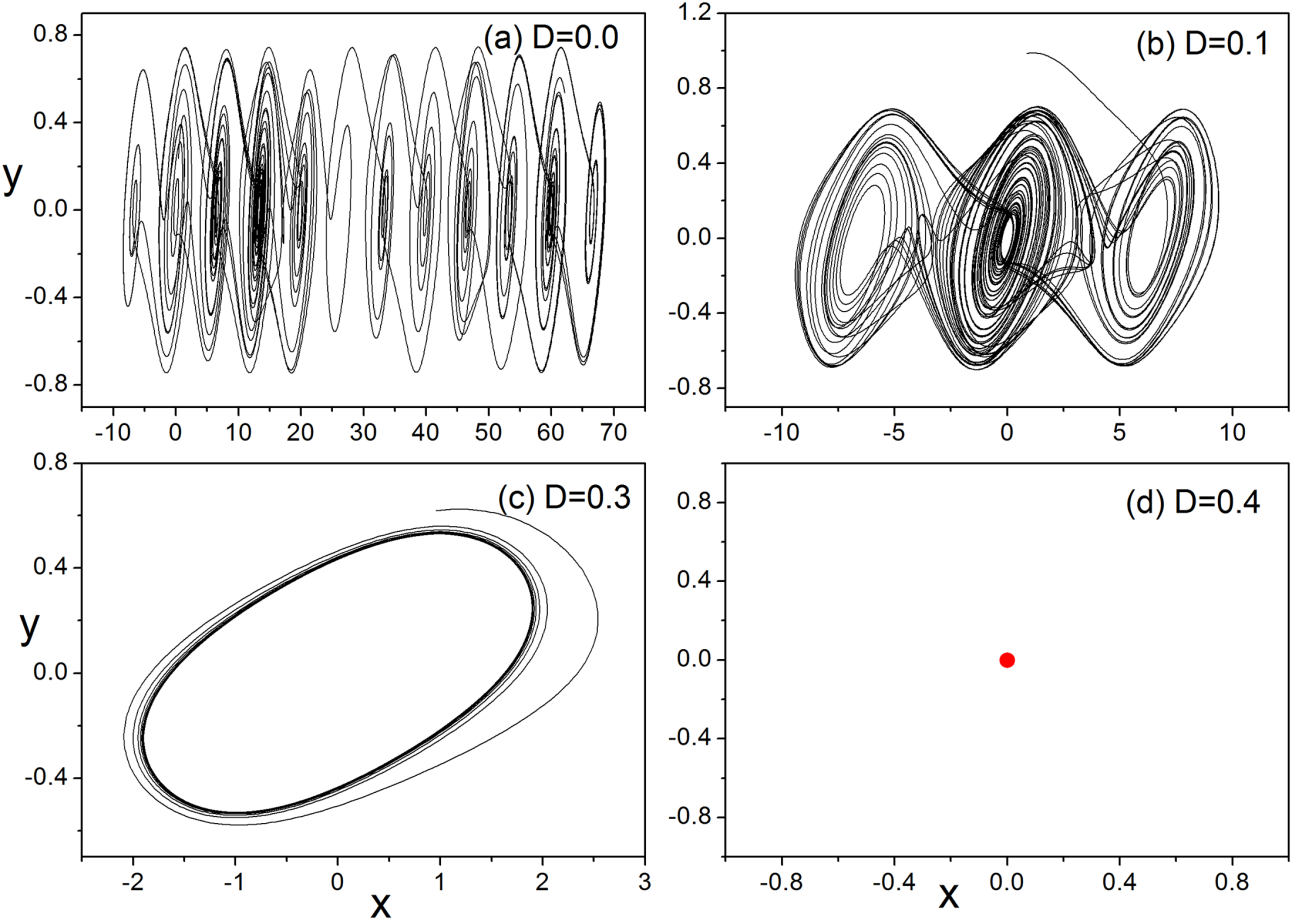
The attractors of the system with different feedback gains. For (a)*D* = 0.0, (b)*D* = 0.1, (c)*D* = 0.3, (d)*D* = 0.4.

The isolated system under negative feedback is described by

$$\left\{ \begin{array}{l} \dot{x}=\alpha (y-a\text{sin}(2\pi bx))-D_{\text{1}}x\\ \dot{y}=x-y+z-D_{\text{2}}y\\ \dot{z}=-\beta y-D_{\text{3}}z \end{array}\right.$$ (12)

where the feedback gains are selected by *D*_1_ = *D*_2_ = *D*_3_ = *D*. Three-channel feedback is considered that three variables (*x*,*y*,*z*) are feedbacked into the isolate circuit, the attractors of the controlled system and bifurcation diagram with different feedback gains are calculated under random initial values in Fig 17.

**Fig 18:**
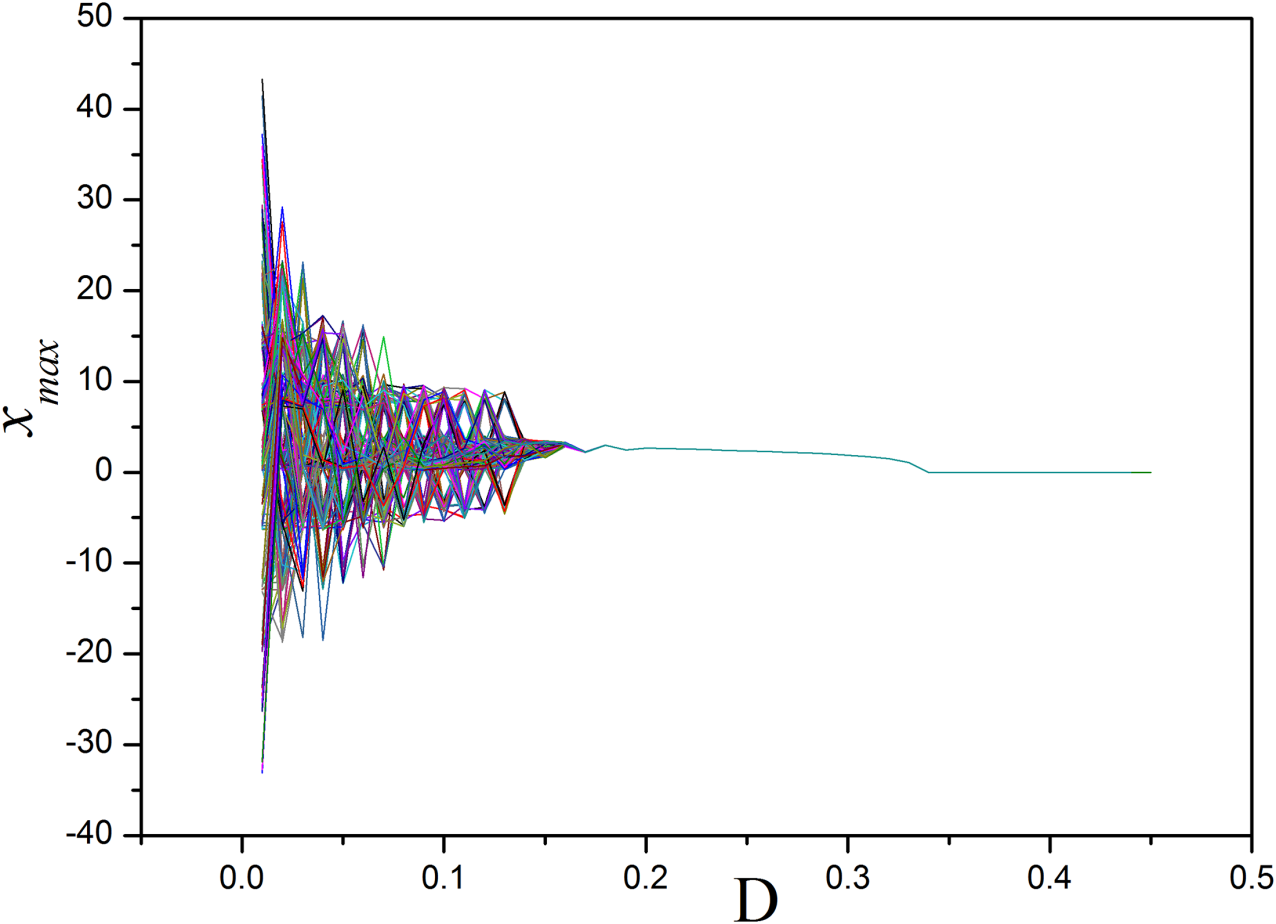
Bifurcation diagram. The bifurcation diagram for the system as a function of feedback gains *D* with three variables (*x*,*y*,*z*) feedback, the random initial values are chosen in the simulation.

The results in Fig 17 confirmed that three-channel feedback on the chaotic circuit makes the system decrease the number of attractors and it even can be stabilized completely. It is also found that multi-channel or multi-variable feedback can control and stabilize the chaotic system effectively than the case for single-channel (or single-variable feedback) and the threshold for feedback intensity can be decreased. Furthermore, the bifurcation diagram for three-channel(or three-variable) feedback is calculated in Fig 18.

The results in Figs 17 and 18 found that the developed state is much dependent on the selection of feedback gain and thus the multi-stability could be adjusted. In a summary, multi-channel or multi-variable feedback, and also multi-channel coupling can be more effective to stabilize the chaotic attractors than single-channel or single-variable feedback or coupling, thus complex spatial patterns could be selected. Therefore, it is also interesting to investigate process of the annihilation of multistability of the system [52,53,54], the basins for an isolated system under different feedback gains are calculated.

To quantify the state for the system at every node in the basin, the region for the multi-scroll attractor in plane (*x*, *y*) is calculated, the scroll area is defined by

**Fig 19:**
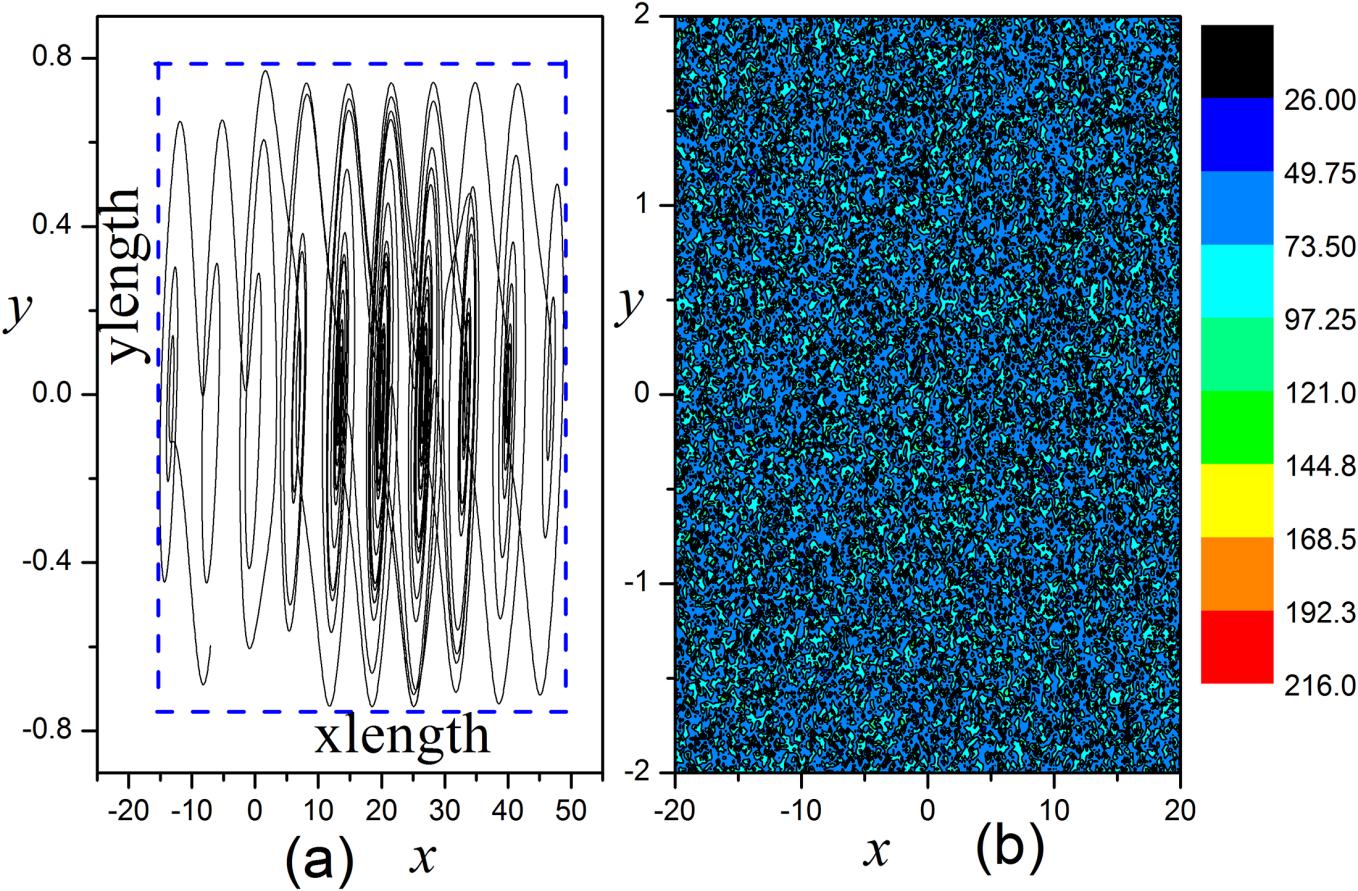
Multi-scroll attractors and basion. (a) The chaotic attractor is developed at *t* = 300 time units, and *D* = 0.0. (b) The basin for the system with *D* = 0.0, initial values of variable *x*, *y* are chosen uniformly in the *x-y* plane box, *x*∈[−20,20], *y*∈[−2,2], z = 0.03.

Area size=xlength×ylength (13)

where *xlength* = *x*_*max*_−*x*_*min*_, *ylength* = *y*_*max*_−*y*_*min*_, *x*_*max*_(*y*_*max*_) and *x*_*min*_(*y*_*min*_) stand for the maximum and minimum value of the variable *x*(*y*), respectively. The results in Fig 9(a) show the attractor for the system within 300 time units, and Area size is associated with the scroll number and also the state of the system. During the calculating area size, time series for output variables are used from *t* = 200 to 300 time units. Different initial values are chosen uniformly in the *x-y* plane box *x*∈[−20,20], *y*∈[−2,2], z = 0.03. The basin for the system with no feedback is calculated in Fig 19(b).

**Fig 20:**
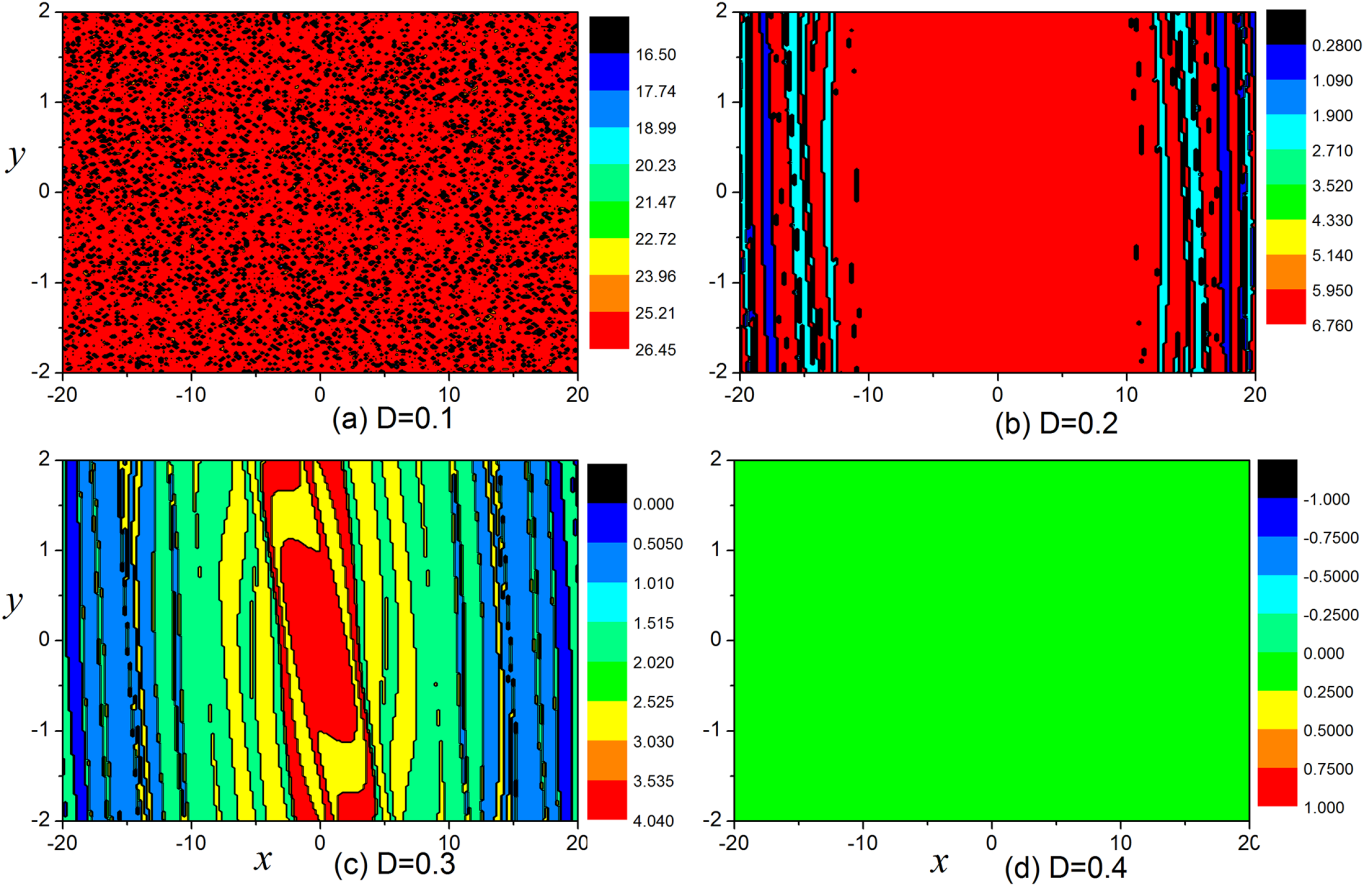
The basin for the system with different feedback gains *D*. For (a) *D* = 0.1, (b) *D* = 0.2, (c) *D* = 0.3, (d) *D* = 0.4, the variables *x* and *y* are selected uniformly in the box [−20,20] and [−2,2].

The distribution of basin in Fig 19 confirmed that the developed states are much dependent on the selection of initial values. Furthermore, it is interesting to investigate the transition of basin and states by applying appropriate feedback gains, and the results are plotted in Fig 20.

The results in Fig 20 show that the multistability is gradually annihilated with increasing feedback gains *D*. In the case of network, the collective behaviors are dependent on the dynamics of each node which can be changed by setting appropriate feedback gain (or coupling intensity). The switch of basin area can induce phase transition of spatial pattern thus different patterns could be formed in the network under appropriate initial values and coupling intensity.

## Conclusions

In this paper, network of coupled multi-scroll attractors is designed to investigate the stability of spatial patterns. It is found that stable spatial pattern can’t be formed in regular connection type. By applying negative feedback with diversity, spiral wave, target wave and homogeneous states could be observed in the network. It indicates that spatial patterns could be stabilized and selected under appropriate feedback gain in diversity and controlled area, the factor of synchronization is calculated to discern the effect of diversity in feedback gain and controlled area. It could present a new challengeable problem and useful guidance for network of multi-scroll attractors.

## Supporting Information

S1 File Supporting data for Fig 20a at D = 0.1. (DAT)

S2 File Supporting data for Fig 20b at D = 0.2. (DAT)

S3 File Supporting data for Fig 20c at D = 0.3. (DAT)

S4 File Supporting data for Fig 20d at D = 0.3. (DAT)
